# The use of MSAP reveals epigenetic diversity of the invasive clonal populations of *Arundo donax* L.

**DOI:** 10.1371/journal.pone.0215096

**Published:** 2019-04-09

**Authors:** Francesco Guarino, Angela Cicatelli, Giuseppe Brundu, Giovanni Improta, Maria Triassi, Stefano Castiglione

**Affiliations:** 1 Department of Chemistry and Biology “A. Zambelli”, University of Salerno, Fisciano, SA, Italy; 2 Department of Agriculture, University of Sassari, Sassari, SS, Italy; 3 Department of Public Health, University of Naples Federico II, Napoli, NA, Italy; Universita degli Studi di Milano-Bicocca, ITALY

## Abstract

Among the most widespread plant species with clonal reproduction *Arundo donax* L. represents one of most studied one characterized by very low genetic biodiversity. Although it is a perennial rhizomatous tall grass native to eastern and southern Asia, it spreads only asexually in the invaded range all over the world thriving very well in a large array of pedo-climatic conditions. This ability to morphologically or physiologically adapt to a broad array of conditions could be attributed to epigenetic mechanisms. To shade light on this relevant issue, 96 stems of *A*. *donax* from spontaneous populations distributed across the Italian invaded range (island of Sardinia, Northern and Southern Italy) were analysed. Leaf DNAs were extracted and processed through AFLPs and MSAPs for defining either genetic and epigenetic profiles. Both analyses clearly showed that the *A*. *donax* populations of Sardinia island are genetically distinct from those of Italian mainland; AFLPs showed an extremely low genetic biodiversity due to vegetative reproduction, whilst, epi-biodiversity, estimated through MSAP marker, increased within the analyzed populations. These results suggest that the capability of *A*. *donax* to invade and thrive in diverse environmental conditions can be, at least, partially attributed to a higher epigenetic variability. Therefore, the different DNA methylation status may have significant and important biological meaning, in particular, in the case of invasive clonal plants such as *A*. *donax*, also for the biodiversity definition, and MSAP marker can be considered an useful and cost effective marker to reveal it.

## Introduction

*Arundo donax* L. (*Poaceae*, subfamily *Arundinoideae*), also known as giant reed, is a perennial rhizomatous tall grass, native to eastern and southern Asia [1, 2]. It has been introduced and grown for local uses in the Mediterranean region since ancient times; traditional uses of *A*. *donax* include basket work, roofing, trellises, musical instruments and traditional medicine, and more recently it started to attract attention in Europe as a potential non-food crop and for biomass production [3]. As a result of this human-mediated spread it is now widely naturalized around the world in areas of North and South America, Asia, Africa, Australia, New Zealand and numerous islands across the Pacific [4]. Although well adapted to widely different ecological conditions, it is basically thrives in riparian habitats, where it forms dense monospecific stands [5, 6]. Its flowers are perfect [7], but neither pollen nor caryopsis have been documented in North America [8] and in other parts of the invaded range (*e*.*g*., for Europe [9]). For this reason, it is considered a sterile plant, which spreads only asexually, through rhizomes at the close proximity of the invaded area and by stem fragments, which can be distributed by water or through human activities at considerable distance from the primary invaded area. Vegetative reproduction is expected to reduce the genetic biodiversity because of absence meiotic mechanisms and, in particular, of its fundamental phases such as crossing over and gene recombination. A relevant number of studies have demonstrated that there is very little genetic variation in *A*. *donax* in the invaded range, *e*.*g*., in Australia [4, 10].

Nevertheless, *A*. *donax* thrives very well in a large array of pedo-climatic conditions around the world, showing different phenotypic and phenological features [11], competing with many native species and displacing native vegetation and arthropod fauna in the invaded sites [12]. This capacity to morphologically or physiologically adapt to a broad array of conditions could be attributed to epigenetics [11]. These mechanisms can expand ecological niche breadth and had been proposed as one potential process involved in plant invasiveness [13]. Studies on clonally spreading plant species, with consequently strong reduction or absence of genetic biodiversity, report that ramets show diverse phenotypes, thrive in diverse environments [11, 14], or face with specific stresses thanks to the presence of epigenetic mechanisms [15–17].

Because of its important function in adaptation, epigenetics may be one of the molecular machineries that drive the biological invasion processes, improving the capability of alien plant species to adapt themselves to (new) ecosystems [18, 19]. Epigenetic variation in newly introduced populations of asexually reproducing species may compensate, to a certain level, for the reduced genetic diversity and serve as an alternative source of phenotypic variation. Changes in DNA methylation has been associated with a number of biological functions (*e*.*g*., pedoclimatic adaptation, promoter inactivation, etc.) and they are thought to be involved in phenotypic plasticity. In particular, symmetrical CpG and CpA(T)pG sites are reported as the most frequent targets for cytosine modification [20], while most genomes also contain 5mC at the outer position of CpCpG sites, albeit at reduced frequencies [21]. These temporary DNA modifications allow phenotypic plasticity, that is an important prerequisite for adaptation, and are often associated with the process of colonization of new environments [22].

*A*. *donax* in its invaded range can be considered an excellent candidate to evaluate and compare biodiversity indices obtained through genetic (AFLP—Amplified Fragment Length Polymorphism) and epigenetic (MSAP—Methylation Sensitive Amplified Polymorphism) markers. In fact, clonal plants represent a suitable *in situ* model for assaying the environmental effects on adaptation capability; because they are sessile organisms, identical from genetic point of view, it is possible to speculate an epigenetic control in the acclimation strategies [23].

Earlier studies, carried out by means of MSAP technique, have drawn attention to the potential role of the environment in shaping phenotypes through methylation of DNA sequences [24, 25]. The results reported by Spens and Douhovnikoff [26] showed a clear tendencies of individual plants toward site- or subspecies- correlated epigenetic fingerprints. Guarino, Cicatelli [27] showed, in the case of white poplar populations, that the DNA methylation status affects the population structure, clustering, and, consequently, influencing the biodiversity indices. Furthermore, Pilu, Cassani [11], studying the *A*. *donax* populations of Sardinia island (Italy), did not exclude the hypothesis that the observed phenotypic differences might be due to epigenetic modifications caused by the particular environment in which the *Arundo* plants thrive.

On the basis of these considerations, *A*. *donax* has been considered a suitable plant species to assay the epigenetic diversity through MSAP marker in its Italian invaded range, characterized by diverse habitats, in comparison with the genetic one analysed through AFLP. To this purpose we used both molecular tools to compare the *A*. *dona*x populations of Sardinia Island previously reported by Pilu et al. [10] as genetically homogenous but thriving in very diverse habitats, and those thriving in mainland Italy.

Therefore, the aims of this study were to: i) compare the genetic structure of the Island specimens versus those thriving in mainland Italy by means of AFLP markers; ii) assay the epigenetic diversity of the populations, characterized by low genetic biodiversity, in response to different habitats.

## Materials and methods

### Study area and collection of plant materials

The leaf collection was conducted in Italy (including the island of Sardinia) in June 2015, during five days with constant meteo-climatic conditions. Leaves from 96 stems of *A*. *donax* were collected across Italy (including the island of Sardinia); the collection sites were geo-referenced using a GPS handy receiver (S1 Table). More precisely, we collected six young leaves (blades) of the same age and size from each of the 96 stems (individuals) of *A*. *donax*, belonging to 14 different populations.

### DNA extraction, AFLP and MSAP analyses, data scoring

Total genomic DNA was isolated from *Arundo* leaves using the DNeasy Plant Mini Kit (Qiagen, Milano-IT). AFLP and MSAP analyses were performed following the protocol of Vos, Hogers [28] and Guarino et al. [2015], respectively.

The AFLP fragments are scored as presence (1) or absence (0) of DNA bands (fragments). All fragments from 50 to 500 bp were considered. The quality of PCR amplifications and bin scoring were assessed as following: DNAs were re-extracted from 10 samples and amplified independently. For the selective amplifications, four primer combinations were used and the error rate, informative of the entire process (restriction-ligation, preselective PCR, selective PCR, scoring), was computed as the sum of errors/total number of comparisons [29]. The average genotyping error rate for each AFLP primer combination was 2.9% below the 10% maximum acceptable error rate proposed by [29].

Regarding the MSAP analysis, the raw data resulting from the *Eco*RI/*Hpa*II and *Eco*RI/*Msp*I profiles were transformed separately into two binary data matrices (with about 170 fragments per matrix), and after that, into a unique binary data matrix, allowing statistical analyses and computation of selected descriptive indices. Although diverse scoring approaches of MSAP profiles are possible [30], for the comparison of *Eco*RI/*Hpa*II and *Eco*RI/*Msp*I fragment profiles, methylation status of the restriction sites were recognised as reported in Table 1. Briefly, the DNA methylation status (5′-CCGG target) was estimated on the basis of the presence/absence of fragments obtained from each enzymatic reactions: the presence of both *Eco*RI/*Hpa*II and *Eco*RI/*Msp*I fragments (pattern 1/1) indicated a un-methylated status; the presence of fragment only in the case of *Eco*RI/*Hpa*II (pattern 1/0) denoted an hemi-methylated CHG-sites (hemi-methylation of inner and outer cytosine); the presence of the fragment only in the case of *Eco*RI/*Msp*I (pattern 0/1) fragments represented a methylated status (double strand methylation of inner cytosine or hemi-methylation of the inner one); the absence of both *Eco*RI/*Hpa*II and *Eco*RI/*Ms*pI fragments (0/0) was considered as uninformative state caused either by different types of methylation or due to restriction site polymorphism (Table 1 –[31, 32]).

**Table 1:** Restriction enzyme behaviour: *Msp*I and *Hpa*II sensitivity to methylation at cytosines in their recognition target.

*Hpa*II	*Msp*I	Methylation status
1	1	No methylation
1	0	Hemi-methylated CHG-sites (Hemi-methylation of inner and outer cytosine)
0	1	Double strand methylation of inner cytosine or hemi-methylation of inner cytosine
0	0	Un-informative state caused either by different types of methylation or due to restriction site polymorphism

Restriction enzyme behaviour in function of full and/or double strand (or hemi-) methylation of inner and/or outer cytosine, 1 indicates the presence of fragment while 0 the absence.

As for AFLP markers, the average error rates for each primer combination (list of primers in S2 Table) was calculated for *Eco*RI/*Msp*I and *Eco*RI/*Hpa*II markers, and was 3.1% and 2.8%, respectively.

### Biostatistical analysis

#### DNA methylation analyses

The *msap* package, available in the R environment (R Core Team 2015 - https://www.R-project.org/), was used to analyse MSAP data matrices (single or unique) and to assess *Msp*I and *Hpa*II differences among groups of specimens [33]. Significant differences between the relative CG and CNG methylation levels and significant differences between the relative total methylation and non-methylation levels were estimated by a Wilcoxon rank sum test within each population. The relative CG, CNG methylation and non- methylation levels among naturalized populations of *A*. *donax* were examined by a Kruskal–Wallis H test. To assess the epigenetic diversity (H) in naturalized populations of *A*. *donax* of Sardinia and the mainland Italy the Shannon’s diversity index (I) was calculated.

### Biodiversity indices: AFLP and MSAP analyses

AFLP (*Eco*RI/*Mse*I) and MSAP (*Eco*RI/*Msp*I and *Eco*RI/*Hpa*II) profiles underwent statistical analyses in order to estimate the common biodiversity indices. Although we know that MSAP profiles are affect by DNA methylation changing, we analysed these profile ignoring that, in order to assay how the biodiversity indices varied between AFLP (genetic analysis) and MSAP (epigenetic analysis); in fact, it is well known that the digestions with isoschizomers *Msp*I and *Hpa*II give different information about the DNA methylation status (Table 1) [31, 32]

One of our aims was to compare the genetic biodiversity, calculated considering the *Eco*RI/*Mse*I indices, with those calculating with *Msp*I and *Hpa*II data sets. The biodiversity indices [number of bands, number of bands with frequency > = 5% (number of different bands with a frequency > = 5%), number of private bands (number of bands unique of each single population), number of locally common bands (frequency > = 5%) found in 25% or fewer populations, number of locally common bands (frequency > = 5%) found in 50% or fewer populations, mean expected heterozygosity (He), with its standard errors] were calculated using Arlequin [34] and GeneAlex software packages [35, 36]. In addition, molecular variance analysis (AMOVA) was performed aiming to estimate inter- and intra- population biodiversity using 9999 permutations of the F*st* value following the methods of Excoffier, Smouse [36], Michalakis and Excoffier [37] and Peakall, Smouse [38]. The binary matrices were elaborated using Jaccard’s distance coefficient (JDC) [39]. Cluster analyses were performed on the similarity matrix by means of the Unweighted Pair Group Mean with Arithmetical Averages (UPGMA) method and using JDC with thousands of bootstrap samples generated by randomly sampling elements of the data. Bootstrap replicates of the dendrogram are obtained by repeatedly applying the cluster analysis to them.

The multivariate AFLP or MSAP datasets were further analysed by Principal Coordinate Analysis (PCoA) using a covariance matrix. The major axes of variation in each dataset were identified and the assessments of the main trends in the data were graphically represented in a reduced-dimension space (*e*.*g*., two dimension scatterplots).

A Bayesian approach was applied, using Structure ver. 2.2 [40], to infer the population structure using the genetic and epigenetic profiles, analysing *Eco*RI/*Mse*I, *Eco*RI*/Msp*I and *Eco*RI*/Hpa*II profiles independently in order to assess whether different methylation levels reflected any population structure. In fact, comparing the probability of the same specimens (obtained from three diverse profiles) to belong, partially or fully, to one or more populations under investigation, it is possible to speculate if and how the DNA methylation status alters the population structure. The number of populations (K) was estimated by performing 10 runs for each population, from K = 1 to K = 10. Each run consisted of 100,000 MCMC (Markov Chain Monte Carlo) permutations with a burn period of 10,000, assuming no *a priori* information on population affiliation, the admixture and correlated allele frequency methods. The K values were estimated using the method of Evanno, Regnaut [41] with 20 independent runs for each K-value [42].

To quantify the differences between the genetic distances, calculated on the basis of the AFLP (*Mse*I profile), and of the MSAP (*Msp*I and *Hpa*II profiles), the following subtractions between genetic distance (GD) matrices were performed: GDMSP-GDAFLP; GDHPA-GDMSP; GDHPA-GDAFLP. It might happen that two distances are roughly similar (resulting in values around zero); other cases where the AFLP or *Msp*I distances are greater than those of *Msp*I or *Hpa*II, showing somewhat smaller values, or, if the opposite is true (GDMSP>GDAFLP, GDHPA>GDMSP or GDHPA>GDAFLP), positive values would be obtained.

## Results

The ninety-six specimens of *A*. *donax*, collected in Italy from 14 populations were analyzed using AFLP and MSAP molecular markers. The AFLP profiles provided molecular data useful for estimating the biodiversity and the population structure in relation to genetic diversity, while, the MSAP profiles (*Eco*RI/*Msp*I and *Eco*RI/*Hpa*II) reflected DNA methylation variability.

### AFLP results

The main biodiversity indices, calculated for the AFLP profiles, demonstrated that the genetic biodiversity of the 14 investigated populations of *A*. *donax* was very low (Table 2); in particular, the number of private bands for each population was zero, and in only two cases equal to three, *i*.*e*. for Pop9 and Pop13. The highest number of common bands (< = 50%) was found in the populations collected in mainland Italy (from Pop8 to Pop13); in contrast, in the other populations (from Pop1 to Pop7 and Pop14), they were absent or close to zero. When the number of common bands (< = 25%) was considered, a maximum value of four was reached within Pop10 and Pop13, three within Pop9, two within Pop12, and one within Pop8 and Pop14.

**Table 2:** Genetic indices calculated for the AFLP pattern.

Population	No. Bands	No. Bands Freq. > = 5%	No. Private Bands	No. LComm Bands (< = 25%)	No. LComm Bands (< = 50%)	Mean He	SE of Mean He	I	SE of I
**Pop1**	81	81	0	0	1	0.000	0.000	0.000	0.002
**Pop2**	82	82	0	0	0	0.004	0.004	0.005	0.006
**Pop3**	82	82	0	0	0	0.022	0.009	0.031	0.013
**Pop4**	81	81	0	0	0	0.001	0.001	0.003	0.003
**Pop5**	82	82	0	0	0	0.004	0.004	0.005	0.006
**Pop6**	83	83	0	0	1	0.010	0.005	0.014	0.008
**Pop7**	82	82	0	0	0	0.003	0.003	0.005	0.006
**Pop8**	84	84	0	1	4	0.013	0.006	0.019	0.010
**Pop9**	109	109	0	3	31	0.000	0.000	0.000	0.000
**Pop10**	115	115	3	4	32	0.044	0.011	0.065	0.016
**Pop11**	102	102	0	0	26	0.000	0.000	0.000	0.003
**Pop12**	109	109	0	2	30	0.000	0.000	0.000	0.003
**Pop13**	115	115	3	4	32	0.038	0.011	0.056	0.000
**Pop14**	87	87	0	1	5	0.022	0.008	0.032	0.010

Abbreviations: No, number; Mean He, mean expected heterozygosity (He) with its standard errors (SE); I, Shannon index with its SE.

In general, the Shannon index (I) was very low (equal or close to zero for all populations–p value > 0.05); only in the case of Pop10 and Pop13, which were collected in Emilia Romagna (mainland Italy), it was 0.065 and 0.056, respectively. However, the molecular variance calculated among populations, equal to 89%, was much higher than that estimated within populations, equal to 11%. This result was due to a higher number of polymorphic loci found in the Pop8, 10, 13, and 14 (3%, 12%, 9%, and 5%, respectively) with respect to those obtained by analysing all the other populations, which showed a percentage of polymorphic loci equal or close to zero.

**Fig 1:**
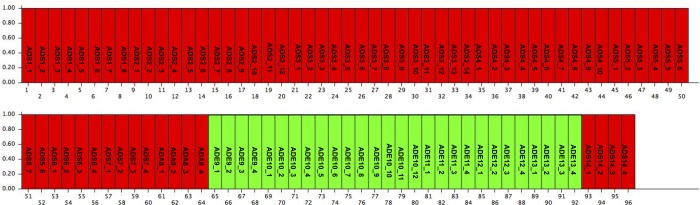
**Bar plot of estimated membership probability (Q) for K = 2 (A) for the AFLP data.** Sample numbers are indicated on the X axis. The estimated membership probability (Q) for K = 2.

To investigate the whole Italian population of *A*. *donax*, Bayesian analyses were performed on the AFLP data set using the Structure software [40]. The delta K criterion indicated K values equal to two (S1 Document). The bar plot for K = 2 is reported in Fig 1: the first cluster included the *A*. *donax* from Sardinia and Campania region (red), whilst, the second, the samples collected in Emilia Romagna region (green).

**Fig 2:**
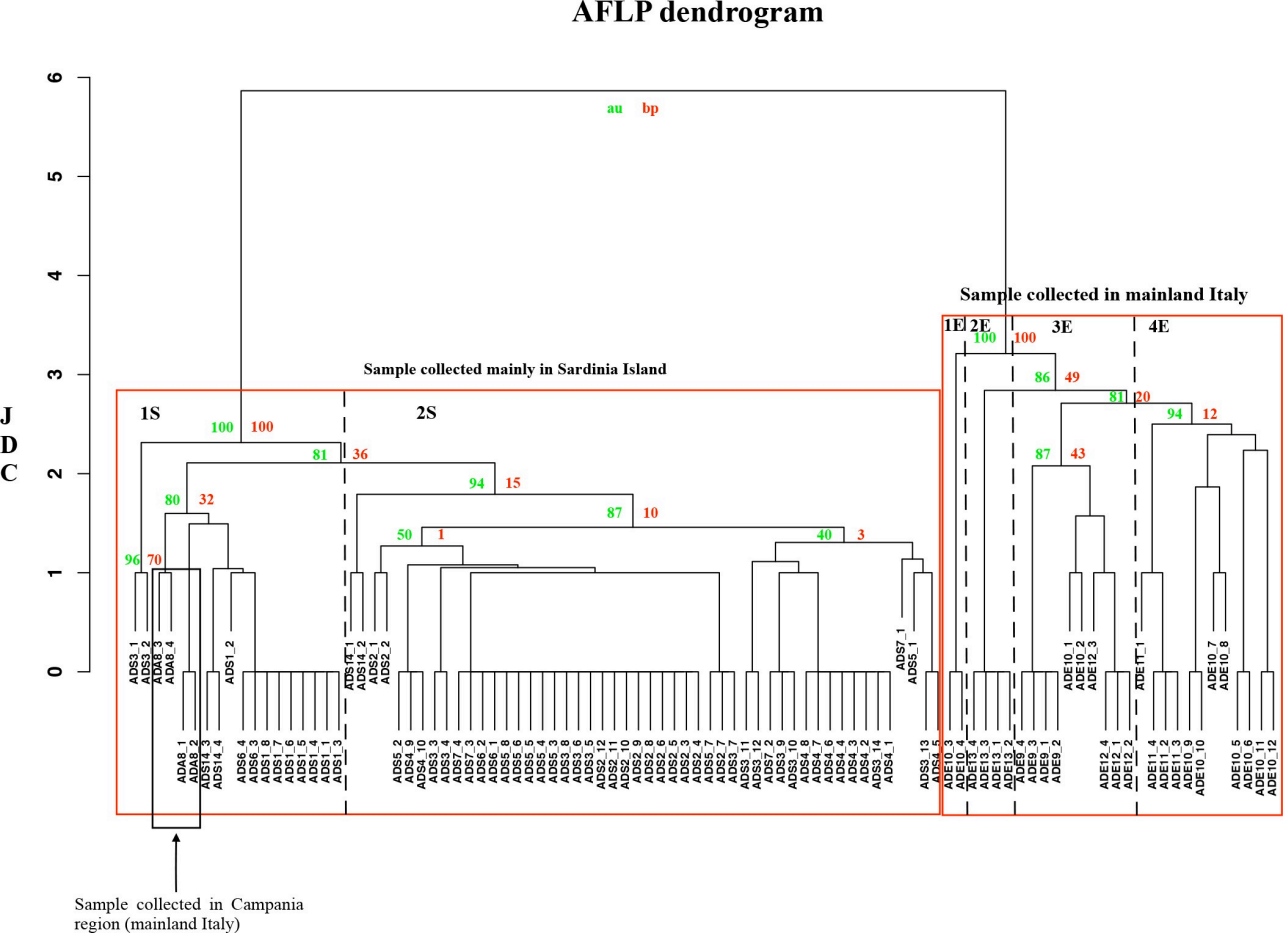
UPGMA dendrogram based on AFLP profiles of *A*. *donax* specimens. The Jaccard Dissimilarity Coefficient (JDC) is indicated on the X axis. The sample names are reported on the Y axis. Download the high definition figure in order to visualize the bootstrap value.

In order to estimate the genetic similarity among the analysed specimens of *A*. *donax*, Jaccard Dissimilarity Coefficient (JDC) was calculated, and the output UPGMA bootstrap dendrogram (Fig 2) confirmed the results obtained through Structure analysis. The specimens collected in mainland Italy and in Sardinia island were clearly separated into two clusters. In the case of the Sardinian samples, they were separated in two subgroups (JDC≅ 2), and included the specimens collected in Campania region (ADA): the first one (1S) included the samples from ADS1_1 to ADS1_8, ADS3_1, ADS3_2, from ADS6_3 to ADS6_4, from ADA8_1 to ADA8_4 and from ADS14_3 to ADS14_4; the second one (2S) included all the others collected in Sardinia. In the case of *A*. *donax* collected in Emilia Romagna, the specimens were subdivided into four subgroups (JDC≅ 3) including: 1E) samples ADE10_3 and ADE10_4; 2E) samples from ADE13_1 to ADE13_4; 3E) sample from ADE9_1 to ADE9_4, ADE10_1, ADE10_2, and from ADE12_1 to ADE12_4; 4E) samples from ADE10_5 to 10_12, and ADE11_1 to ADE11_4. The highest JDC was reached in the case of the node that grouped samples ADE10_3 and ADE10_4, which can be considered, therefore, as an out-group. However, the AFLP analyses confirm that *A*. *donax* adopts vegetative reproduction and, in particular, the population are constituted by mono-clonal stands.

### MSAP results

The ability of *A*. *donax* to invade and thrive in diverse non-native habitats, and in Sardinia in particular, and the fact that it shows different phenotypes and phenology in clonal populations [11, 43], may be due to epigenetic variations. We therefore investigated DNA methylation status using MSAP.

### The “msap” analysis

The “msap” algorithm, developed in the R environment [28], was used to estimate the cytosine methylation status of the *A*. *donax* populations. Given that the specimens collecting from the 14 populations shown very similar AFLP genetic profiles and that the statistical analyses divided all the samples in just two groups (meta-populations), we fixed the *a priori* number of groups equal to two for the analyses performed on MSAP data, in agreement with the estimated number of groups obtained by the Structure software and UPGMA analyses for AFLP data.

**Fig 3:**
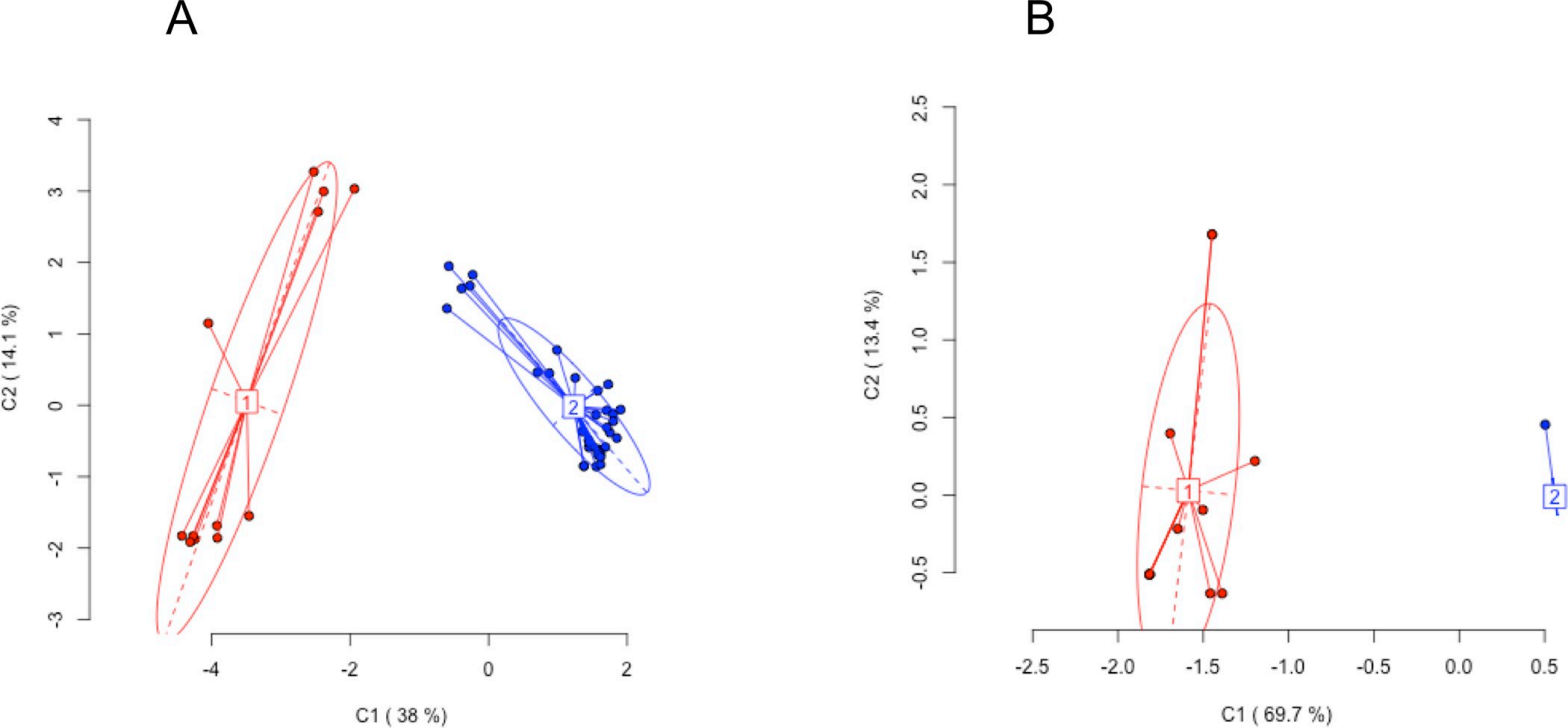
Representation of Principal Coordinate Analysis (PCoA) for epigenetic differentiation among groups. Different colours represent the two different meta-populations, while the dots indicate Arundo samples. The numbers 1 and 2 indicate the position of the genetic centroids. Ellipses represent the average dispersion of those individual data points around their centre. The long axis of the ellipse shows the direction of maximum dispersion and the short axis, the direction of minimum dispersion. Fig 3A and 3B show *Msp*I and *Hpa*II results, respectively.

The Principal Coordinate Analysis (PCoA; Fig 3), calculated using the *Msp*I (Fig 3A) or *Hpa*II profiles (Fig 3B), showed that the epigenetic diversity within the analysed populations was larger when *Hpa*II profiles were considered.

The *Msp*I PCoA (Fig 3A) showed that the two meta- populations (red and blue) although well separated, showed a greater degree of dispersion within each ellipse. However, in the case of *Hpa*II PCoA (Fig 3B), the two meta-populations were still separated with a lower dispersion intra-population.

The DNA methylation status was estimated for all the 14 geographic populations; double strand methylation of inner cytosine, or its hemi-methylation (27%, for both the populations) was the most abundant methylation status.

In order to estimate the biodiversity due to different DNA methylation status, the Shannon diversity index was calculated separately for *Hpa*II and *Msp*I profiles. The results obtained were 0.51 and 0.35, respectively. A Wilcoxon rank sum test with continuity correction was significant (P<0.01). The percentage of polymorphic methylation sensitive loci, revealed by *Msp*I and *Hpa*II digestions, was 15% and 90%, respectively.

### Biodiversity indices

A new approach was applied to analyse the MSAP data; until now the two profiles obtained during the MSAP analysis (*Msp*I and *Hpa*II) have been processed together, as we made above. However, in our opinion, in the case of clonal populations, it could be possible to consider the two profiles independent; in this way each difference in the biostatistical indices will be attributed to DNA methylation effect, or to error rate. The main biodiversity indices are reported in Tables 3 and 4.

**Table 3:** Genetic indices calculated for the *Msp*I pattern.

Population	No. Bands	No. Bands Freq. > = 5%	No. Private Bands	No. LComm Bands (< = 25%)	No. LComm Bands (< = 50%)	Mean He	SE of Mean He	I	SE of I
**Pop1**	138	138	0	0	1	0.014	0.006	0.020	0.008
**Pop2**	142	142	2	14	17	0.068	0.012	0.100	0.017
**Pop3**	154	154	1	14	17	0.163	0.016	0.235	0.022
**Pop4**	137	137	0	0	0	0.028	0.008	0.040	0.014
**Pop5**	139	139	0	0	3	0.017	0.006	0.025	0.012
**Pop6**	138	138	0	0	1	0.010	0.005	0.014	0.006
**Pop7**	138	138	0	1	2	0.007	0.004	0.011	0.007
**Pop8**	139	139	0	1	1	0.010	0.005	0.014	0.006
**Pop9**	139	139	0	0	1	0.015	0.006	0.022	0.007
**Pop10**	140	140	0	0	2	0.037	0.010	0.053	0.012
**Pop11**	136	136	0	0	0	0.000	0.000	0.000	0.011
**Pop12**	135	135	0	0	3	0.000	0.000	0.000	0.006
**Pop13**	138	138	0	0	19	0.010	0.005	0.014	0.007
**Pop14**	147	147	0	16	0	0.108	0.014	0.158	0.021

Abbreviations: No, number; Mean He, mean expected heterozygosity (He) with its standard errors (SE)); I, Shannon index with its SE.

**Table 4:** Genetic indices calculated for the *Hpa*II pattern.

Population	No. Bands	No. Bands Freq. > = 5%	No. Private Bands	No. LComm Bands (< = 25%)	No. LComm Bands (< = 50%)	Mean He	SE of Mean He	I	SE of I
**Pop1**	121	121	0	0	1	0.013	0.006	0.020	0.008
**Pop2**	141	141	2	2	16	0.141	0.016	0.206	0.022
**Pop3**	137	137	1	2	13	0.088	0.013	0.130	0.018
**Pop4**	125	125	0	1	3	0.097	0.015	0.137	0.020
**Pop5**	122	122	0	0	1	0.015	0.006	0.022	0.008
**Pop6**	123	123	0	0	3	0.020	0.007	0.029	0.007
**Pop7**	130	130	0	1	6	0.074	0.012	0.109	0.009
**Pop8**	123	123	0	1	5	0.056	0.011	0.081	0.023
**Pop9**	129	129	0	2	27	0.057	0.011	0.083	0.005
**Pop10**	131	131	0	2	28	0.109	0.014	0.161	0.021
**Pop11**	112	112	0	0	22	0.050	0.011	0.071	0.014
**Pop12**	131	131	0	4	30	0.064	0.012	0.094	0.008
**Pop13**	142	142	2	5	33	0.091	0.013	0.137	0.000
**Pop14**	120	120	0	0	0	0.009	0.005	0.014	0.004

Abbreviations: No, number; Mean He, mean expected heterozygosity (He) with its standard errors (SE); I, Shannon index with its SE.

The number of private bands in each of the 14 populations was zero in the case of the *Msp*I data set, with the exceptions of Pop2 and Pop3, which showed a value equal to two and one, respectively. In contrast, when the *Hpa*II profile was considered, this value was two for Pop2 and Pop13, and one for Pop3. The number of common bands was in general very low, with the exception of the Pop2, Pop3 and Pop13; in fact, they showed the highest number of common bands (< = 50%) for *Msp*I profile. In the case of *Hpa*II, Pop2, Pop3, and populations from 9 to 13 showed the highest number of common bands (< = 50%); the highest number of common bands was detected in Pop13. Nevertheless, the percentage of polymorphic bands ranged between 0.0% (Pop11 and 12) and 39.0% (Pop3) in the case of the *Msp*I profile, and between 0.0% (Pop11 and 12) and 51.0% (Pop3) in the case of *Hpa*II, and, in general, it increased when the latter profile was considered. The inter- and intra-population molecular variance was 53% and 47% for *Msp*I profile, and 58% and 42% for *Hpa*II profile.

The genetic structure of the 14 Italian *A*. *donax* populations was analysed with no *a priori* information, using the Structure software [40] for both data sets (*Msp*I or *Hpa*II). The statistical model described by Evanno, Regnaut [41] showed two peaks for *Msp*I at the *K* value two and three (S2 Document), and one for *Hpa*II data sets at K value equal to three (S3 Document).

**Fig 4:**
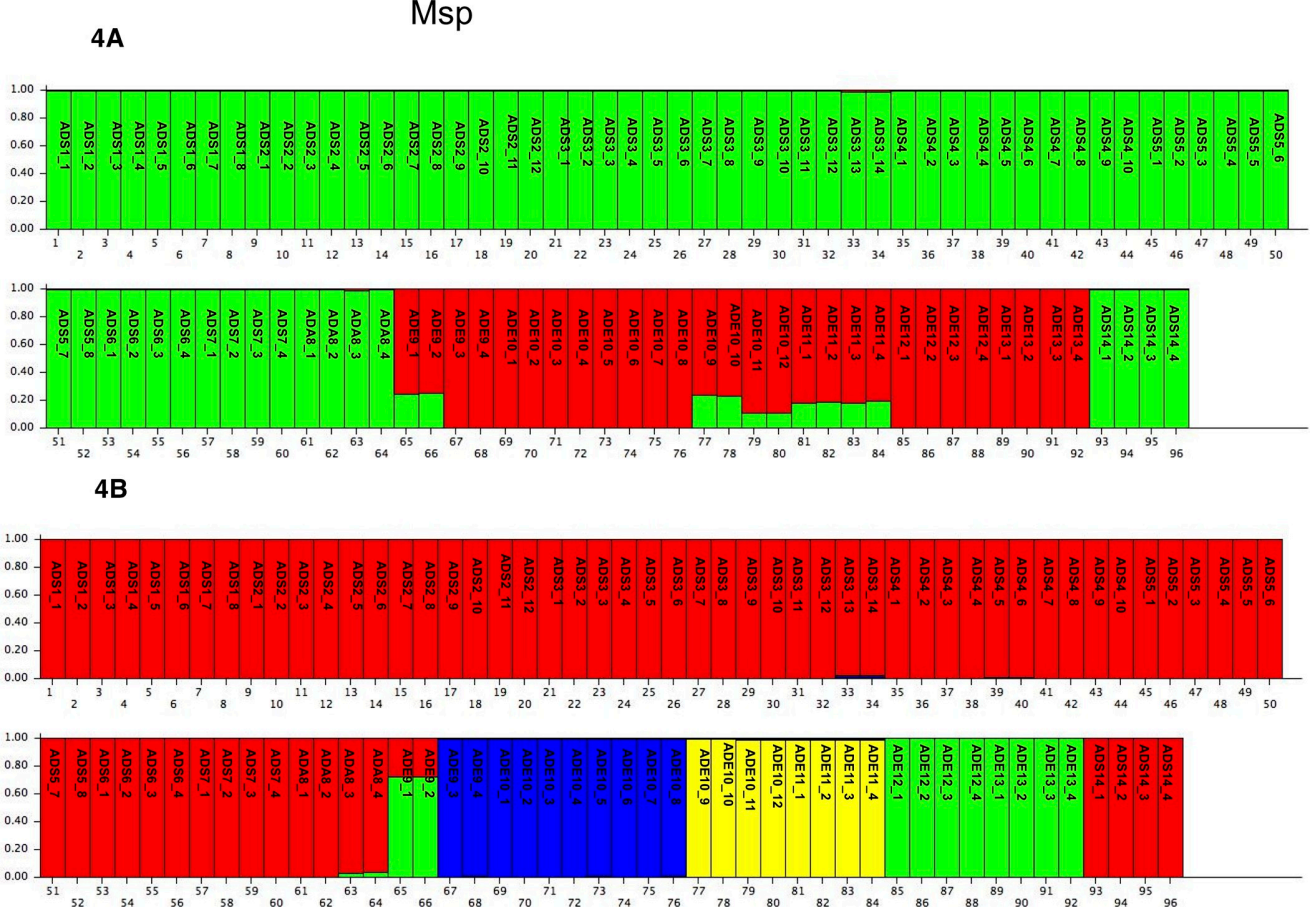
**Bar plot of estimated membership probability (Q) for K = 2 (A) and K = 3 (B) for the *Msp*I profiles.** Sample numbers are indicated on the X axis. The estimated membership probability (Q) for K = 2 (A) and K = 3 (B) are represented on the Y axis.

The bar plot obtained for the *Msp*I data set in the case *K* = 2 (Fig 4A) was quite similar to that reported for the AFLP data. A single cluster included all of the *A*. *donax* specimens collected in Sardinia and in Campania (green bars), and the other cluster included all of the other *A*. *donax* specimens collected in mainland Italy (red bars). In contrast to the AFLP result, ten specimens, collected in Emilia Romagna region (ADE9_1, ADE9_2, and from ADE10_9 to ADE11_4), were assigned both to red cluster (with the probability, roughly 80%) and to green cluster (with the probability of roughly 20%). When K = 4 was considered (Fig 4B), the Sardinian specimens showed the population structure similar to K = 2, whilst the *A*. *donax* collected in Emilia Romagna region were divided amongst three different clusters. In particular, the specimens from ADE9_3 to ADE10_8, were separated from all the other ADE individuals and assigned to the blue cluster; the specimens from ADE10_9 to ADE11_4 (that in the case of K = 2 did not show a clear membership) were assigned to the yellow one; the specimens from ADE12_1 to ADE13_4 constituted the green cluster.

**Fig 5:**
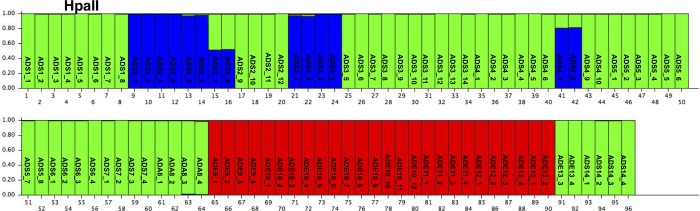
Bar plot of estimated membership probability (Q) for K = 3 for the *Hpa*II profiles. Sample numbers are indicated on the X axis. The estimated membership probability (Q) for K = 3 are represented on the Y axis.

The optimal population number and the cluster membership changed when the *Hpa*II profile was considered. In this case, K was equal to three, and several specimens showed an ambiguous membership (Fig 5). In the case of *Hpa*II, the *A*. *donax* specimens from Sardinia, which by AFLP and *Msp*I analyses belonged to the same cluster, were here split into two clusters: green and blue. In particular, the specimens from ADS2_1 to ADS2_6 were clearly assigned to the blue cluster, as well as samples from ADES3_1 to ADS3_4, whilst all the other *A*. *donax* of Sardinia island belonged to the green cluster, with few exceptions: ADS2_7, ADS2_8 showed 50% of probability to belong to the blue cluster; ADS4_7 and ADS4_8 showed a low probability (about 20%) to be assigned to the green one. Furthermore, the samples collected in Emilia Romagna were clearly assigned to the red cluster.

**Fig 6:**
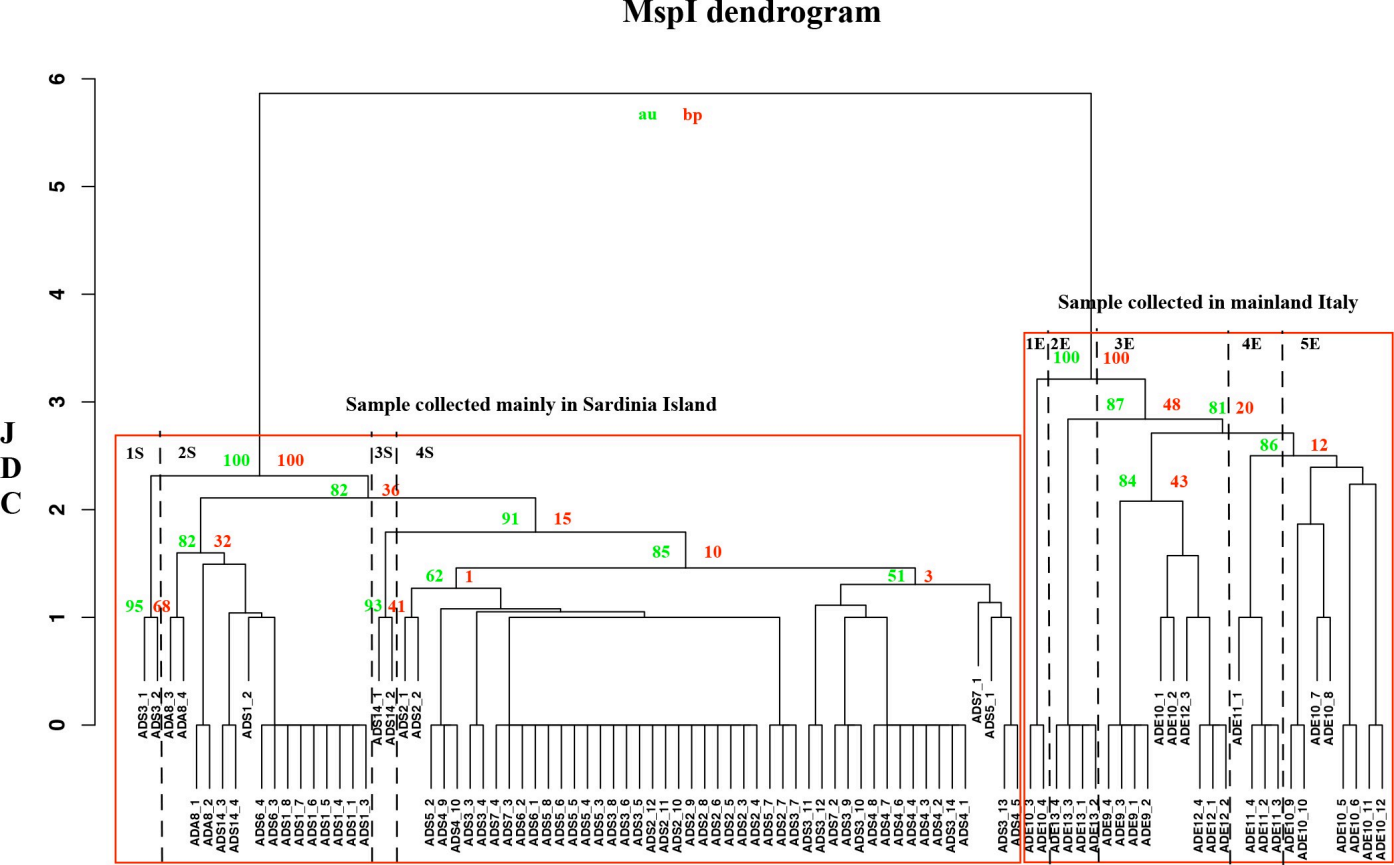
UPGMA dendrogram based on *Msp*I profiles of *A*. *donax* specimens. The Jaccard Dissimilarity Coefficient (JDC) is indicated on the X axis. The sample names with their previously assigned (or not) genotype are reported on the Y axis. Download the high definition figure in order to visualize the bootstrap value.

**Fig 7:**
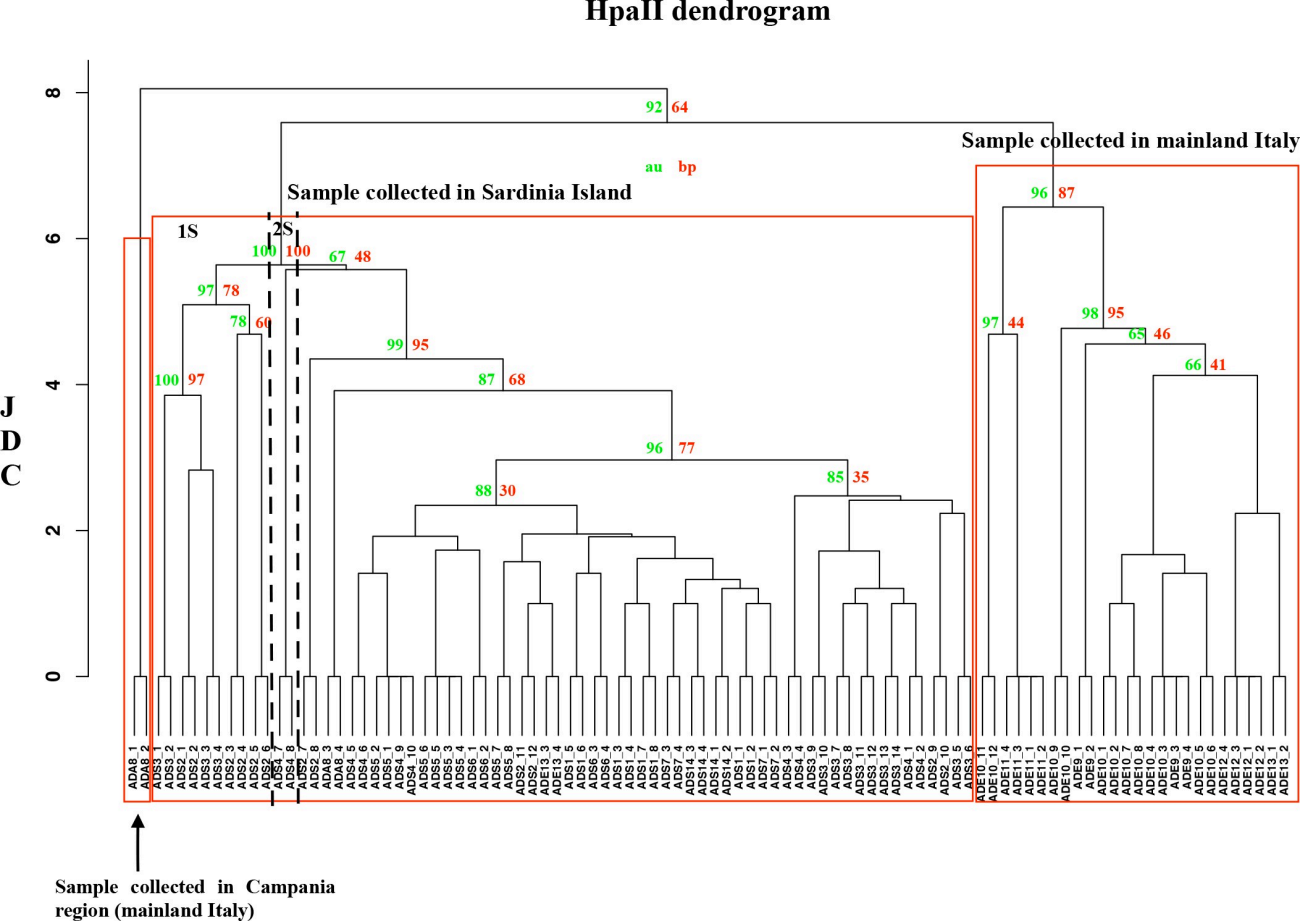
UPGMA dendrogram based on *Hpa*II profiles of *A*. *donax* specimens. The Jaccard Dissimilarity Coefficient (JDC) is indicated on the X axis. The sample names with their previously assigned (or not) genotype are reported on the Y axis. Download the high definition figure in order to visualize the bootstrap value.

In order to visualize the level of similarity among the *A*. *donax* specimens, the JDC was calculated for both *Msp*I and *Hpa*II data sets, and the two UPGMA dendrograms are reported in Figs 6 and 7, respectively. The DNA methylation status revealed by the *Msp*I and *Hpa*II profiles showed an increased epigenetic diversity when compared to those determined by AFLP. In the case of *Msp*I profile (Fig 6), we found two cluster with a p value lower than 0.05 related to the groups of Sardinia Island and mainland Italy. However, in the dendrogram is possible identify other subgroups. In the case of *A*. *donax* collected in Sardinia Island plants are separated in 4 clusters with the highest JDC ≅ 2: 1S) sample ADS3_1 and ADS3_2; 2S) from ADS1_1 to ADS1_8, ADS6_3 and ADS6_4, ADS8_1 to ADS8_4, and ADS14_3 and 14_4. In the case of *A*. *donax* collected in Emilia Romagna, the specimens were subdivided into five subgroups (JDC≅ 3) including: 1E) samples ADE10_3 and ADE10_4; 2E) from ADE13_1 to ADE13_4; 3E) ADE9_1 to ADE9_4, ADE10_1 and ADE10_2, from ADE12_1 to ADE12_4; 4E) from ADE11_1 to ADE11_4; 5E) from ADE10_5 to ADE10_12.

In the case of *Hpa*II profile (Fig 7), the number of clusters with a p value lower than 0.05 were equal to three, highlighted in Fig 6 by three square with red contour, and related respectively to: sample (ADA8_1 and ADA8_2) collected in Campania region (mainland Italy), sample (ADS) collected in Sardinia Island, and those (ADE) of Emilia Romagna region (mainland Italy). In these three cluster, statistically significative, it is possible to identify some subgroups: 1S) from ADS2_1 to ADS2_6 and ADS3_1 to ADS3_4; moreover, the membership of each cluster changed, in agreement with the results obtained using Structure analysis.

To quantify the differences between the genetic distances, the following subtractions between genetic distance (GD) matrices were performed: GDMSP-GDAFLP; GDHPA-GDMSP; GDHPA-GDAFLP (as reported in section 2.2). In our study, the GDHPA was greater than that calculated in the case of AFLP and *Msp*I. In particular, GDHPA was greater than GDAFLP for all specimens (highlighted in green) (S3 Table). A similar result, albeit less pronounced, was obtained comparing GDMSP and GDAFLP (S4 Table). In this case, the differences appeared to be neutral or close to neutrality (highlighted in pale green or in blank, respectively) for a large part of the specimens analysed, with the exception of some samples collected in Emilia Romagna (S4 Table). When GDMSP was subtracted to GDHPA (S5 Table), the GDHPA matrix was in general greater than that of GDMSP.

## Discussion

Plants in general, and in particular the invasive riparian one, may have developed molecular processes to adapt themselves in order to colonize new and frequently changing habitats. Epigenetic mechanisms are, very likely, the trigger of a series of complex molecular mechanisms that allow plants to thrive in sometimes harsh conditions. In order to understand whether epigenetic biodiversity, rather than genetic diversity, might better explain the invasive success of the archaeophyte *A*. *donax* in Italy, we analysed 96 individuals from 14 naturalized populations using AFLP (*Eco*RI/*Mse*I–genetic diversity) and MSAP (*Eco*RI/*Msp*I and *Eco*RI/*Hpa*II–epigenetic diversity).

In general, plants use epigenetic mechanisms to counteract environmental variations and habitat changes [44], or cope with various stress factors, such as soil or water pollution, season whether conditions, etc. [15, 16, 45]; moreover, several studies have reported the presence of new and different phenotypes in clonal plants, and, also in animals.

Different epi-phenotypes, although related to the same genome, are hypothesized to promote significantly environmental adaptation, because they promote phenotype diversification and increase the probability to win the “war for life” in new or highly competitive ecosystems, or in relation to environment changes. The benefits of phenotype diversification induced by epigenetic variation are particularly noticeable in plant species that adopt vegetative reproduction, which may explain the “invasion paradox” [46], or the fact that exotic species are able to colonize, occupy and supplant native species that are well adapted to that specific habitat.

In order to evaluate the epigenetic status of an invasive alien plant species such as *A*. *donax*, and to compare it with genetic diversity, a set of genetic biodiversity indices, estimated on the basis of AFLP data (genetic profile), were compared with those obtained with MSAP markers (epigenetic profiles). The Jaccard Distance Coefficient (JDC) revealed a very low genetic biodiversity in the case of *A*. *donax* collected in Sardinia (ADS) and in Emilia Romagna (ADE). The AFLP analyses confirmed the particular tendency for vegetative reproduction and, at the same time, demonstrated that the specimens of *A*. *donax* collected in Sardinia are genetically isolated from those thriving in Emilia Romagna. A high similarity value was expected; in fact, Mariani, Cabrini [5], suggesting the monophyletic origin of *A*. *donax*, also asserted that this species, originating in Asia, where it is not sterile [1], spread clonally all over the world, and in particular in the Mediterranean basin. In support of this hypothesis, the authors also demonstrated, using AFLP markers, that the *A*. *donax* populations of the Mediterranean basin are characterized by a very low genetic diversity, and by a low incidence of rare polymorphic fragments. Despite the low genetic biodiversity of *A*. *donax* found in Sardinia and in southern Italy, Pilu, Cassani [11] and Cosentino, Copani [43], respectively, observed a significant phenotypic variability, to which did not correspond a quite similar high genetic biodiversity. In terms of phenology (date of flowering), allometry (stem density, stem height, *etc*.) and biomass production, the results reported by Pilu et al. [10] and Cosentino et al. [39] showed a high morphological diversity that probably allows *A*. *donax* to invade and colonize different environments, although the Italian population of giant reed is characterized by a very limited genetic biodiversity due to primary asexual reproduction and seed sterility [7]. This ability is indeed fascinating as it might open new scenarios for the adaptation of this species and other clonal invasive species to different environments.

Our data are support the hypothesis that epigenetic mechanisms, and in particular DNA methylation status, may be involved in the adaptation of *A*. *donax* to diverse habitats, and might be responsible for the different phenotypes and physiological responses, as highlighted by Pilu, Cassani [11]. This study showed that the genetic biodiversity is not sufficient to explain the differences in terms of phenotype, flowering, etc. Moreover, the biodiversity indices based on morphological aspects may be affected by error due to subjectivity; for this reason, we assayed epigenetic differences that may be due to habitats and/or to any other environmental conditions and that could also modify the plant phenotype and facilitate invasion. Epigenetic diversity has become an issue of great interest for biodiversity studies [27, 47–49]; in fact, it was demonstrated that changes in DNA methylation status can be the molecular tools adopted by plants for adapting themselves to different habitats and external stimuli [10, 50–52]. *A*. *donax* is a good model organism for studies comparing biodiversity and population structure as obtained by AFLP and MSAP because of its vegetative reproduction. The differences observed in the populations studied here, using these two molecular approaches, are probably related to the different environments in which the populations are now thriving. Presumably, as plants cannot move around to find the best habitat, they had to adopt molecular mechanisms, such as epigenetic changes, to counteract diverse external stimuli (*e*.*g*., environmental, biotic stress, etc.) and, consequently, modify gene expression [45, 52, 53–55, 56, 57]. Plant DNA is usually highly methylated (ca. 6%–30% of methylated sites–[58]), but there are large differences in the level of 5mC among species. For example, Wang et al.[59] and Hauben, Haesendonckx [60] found epigenetic polymorphisms in cotyledons and leaves of canola, whilst Teyssier, Bernacchia [61] demonstrated that fruit ripening in the mature leaves and pericarp is affected by alteration of cytosine methylation in tomato plants. In our study, we demonstrated that the genome of *A*. *donax* is highly methylated (ca. 50% of the amplified loci were sensitive to methylation). It is known that epigenetic modifications are crucial for promoting phenotypic variation in living organisms [62–64], as also suggested by the results reported here and by the hypothesis stated by Pilu, Cassani [11]. Epigenetic effects, involved in short- and medium-term adaptation, have been identified for several plant species, including the white and black poplars, mangrove and rice [27, 47, 62, 65], suggesting that plants that reproduce vegetatively will be able to improve their fitness over time despite having low genetic diversity within their populations. For instance, if a pioneer plant was not optimally acclimatized to a specific ecosystem, it might, during its lifetime, produce ramets that are epigenetically different and showing a better fitness [23]. Such a mechanism of acclimatization is similar to that indicated by Fischer, Van Kleunen [66] and Frappier, Lee [67] in the case of the exotic species *Ranunculus reptans* L. and *Rhamnus frangula* L., respectively, to explain the late appearance of the invasive features with respect to the start of their habitat colonization. After each generation, the specimens would be better adapted to the local conditions, independently of their genetic profile [47, 68].

Our results, here reported, shed light on the effects of DNA methylation on biodiversity and on the invasive ability of *A*. *donax*. Bayesian and non-Bayesian analyses showed consistent results for each profile analysed. In particular, when *Hpa*II profiles were considered, the optimal number of populations and the recognized clusters present in the dendrogram were different compared to those obtained using AFLP and *Msp*I profiles. Therefore, the different optimal population numbers underlie diverse memberships on the basis of DNA methylation status and a higher biodiversity. In general, when *Msp*I and *Hpa*II dendrograms were considered, the similarity coefficient decreased because of the different DNA methylation status of each *A*. *donax* specimen. Similar to the bar plot, the AFLP dendrogram well separated ADS and ADE specimens into clusters, although the similarity value and the cluster memberships varied when the two different molecular profiles were considered, revealing a variation of the clustering when compared with AFLP (genetic), or *Msp*I (epigenetic) profiles.

This observation suggests that pedo-climatic conditions may generate variations in the DNA methylation status that drive the mechanisms of convergence and/or divergence of populations experiencing similar/dissimilar habitat; as suggested by Schulz et al. 2014 [29], this may neutralize the effects of historical demographic processes and, probably, increase invasiveness.

In conclusion, our study confirms that the genetic biodiversity of the archaeophyte *A*. *donax*, present in diverse regions in Italy, is very low. Moreover, the populations of Sardinia are genetically isolated and clearly differentiated from those growing in Emilia Romagna, suggesting different introduction routes of this plant species in the two Italian regions. The estimated indices and population structure show that the similarity decreases when *Msp*I and *Hpa*II profiles are considered, and, furthermore, that the population structure is altered by the different DNA methylation status. Our results suggest that the ability of *A*. *donax* to invade and thrive in diverse environmental conditions can be attributed, at least, in part to a higher epigenetic variability as previously hypothesized by Pilu, Cassani [11] in the case of Sardinia populations. Therefore, in our opinion, the MSAP technique represents an efficient and cost-effective tool with which is possible to measure biodiversity at epigenetic level. Moreover, the epigenetic profiles should be considered and added to those commonly employed in the framework of the Convention on Biological Diversity. We foresee that epigenetic profiling could be determined in a similar way to genetic profiling, using the same indices, or developing new ones, but by processing DNAs separately based on methylation-sensitive and insensitive profiles. Therefore, we are convinced that the investigation of the DNA methylation status is fundamental for basic ecological and biodiversity studies, particularly in the case of plant species that propagate vegetatively, as suggested in our previous work [27] and by other authors [23, 26], Guarino, Cicatelli [27, 69, 70].

## Supporting information

S1 Table Geographic coordinates of the collected *A*. *donax* specimens. (DOCX)

S2 Table Table of used primers. (XLSX)

S3 Table Difference between genetic distance matrices calculated for AFLP and *Hpa*II profiles. (XLSX)

S4 Table Difference between genetic distance matrices calculated for AFLP and *Msp*I profiles. (XLSX)

S5 Table Difference between genetic distance matrices calculated for *Msp*I and *Hpa*II profiles. (XLSX)

S1 Document Results of Evanno harvester–Structure analyses. (PDF)

S2 Document Results of Evanno harvester–Structure analyses. (PDF)

S3 Document Results of Evanno harvester–Structure analyses. (PDF)
