# Supplementary material for: The use of MSAP reveals epigenetic diversity of the invasive clonal populations of Arundo donax L
Source: PLoS One. 2019 Apr 9;14(4):e0215096. doi: 10.1371/journal.pone.0215096 (PMC6456200; doi:10.1371/journal.pone.0215096)
Supplement: S1 Table — (DOCX) [file pone.0215096.s001.docx]

Table SM1

| 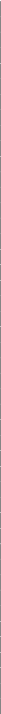   \| Name \| \| --- \| | Longitude | Latitude | Population | Municipality |
| --- | --- | --- | --- | --- | --- |
| ADS1_1 | 9,59127027 | 39,3721815 | 1 | Muravera |
| ADS1_2 | 9,59127027 | 39,3721815 | 1 | Muravera |
| ADS1_3 | 9,56525375 | 39,432628 | 1 | Villaputzu |
| ADS1_4 | 9,56525375 | 39,432628 | 1 | Villaputzu |
| ADS1_5 | 9,61724594 | 39,5149812 | 1 | Villaputzu |
| ADS1_6 | 9,61724594 | 39,5149812 | 1 | Villaputzu |
| ADS1_7 | 9,58642723 | 39,5271247 | 1 | San Vito |
| ADS1_8 | 9,58642723 | 39,5271247 | 1 | San Vito |
| ADS2_1 | 9,58827874 | 39,5273045 | 2 | San Vito |
| ADS2_2 | 9,58827874 | 39,5273045 | 2 | San Vito |
| ADS2_3 | 9,58863606 | 39,5269063 | 2 | San Vito |
| ADS2_4 | 9,58863606 | 39,5269063 | 2 | San Vito |
| ADS2_5 | 9,58888381 | 39,5259319 | 2 | San Vito |
| ADS2_6 | 9,58888381 | 39,5259319 | 2 | San Vito |
| ADS2_7 | 9,60964486 | 39,429196 | 2 | Villaputzu |
| ADS2_8 | 9,60964486 | 39,429196 | 2 | Villaputzu |
| ADS2_9 | 9,60440676 | 39,3176712 | 2 | Muravera |
| ADS2_10 | 9,60440676 | 39,3176712 | 2 | Muravera |
| ADS2_11 | 9,58068994 | 39,3338221 | 2 | Muravera |
| ADS2_12 | 9,58068994 | 39,3338221 | 2 | Muravera |
| ADS3_1 | 8,99603892 | 39,2059366 | 3 | Assemini |
| ADS3_2 | 8,99603892 | 39,2059366 | 3 | Assemini |
| ADS3_3 | 9,38174749 | 39,1656897 | 3 | Quartu Sant'Elena |
| ADS3_4 | 9,38174749 | 39,1656897 | 3 | Quartu Sant'Elena |
| ADS3_5 | 9,29404501 | 39,2168293 | 3 | Quartu Sant'Elena |
| ADS3_6 | 9,29404501 | 39,2168293 | 3 | Quartu Sant'Elena |
| ADS3_7 | 8,99724483 | 39,1710188 | 3 | Capoterra |
| ADS3_8 | 8,99724483 | 39,1710188 | 3 | Capoterra |
| ADS3_9 | 9,00925126 | 39,0959015 | 3 | Sarroch |
| ADS3_10 | 9,00925126 | 39,0959015 | 3 | Sarroch |
| ADS3_11 | 8,87175616 | 38,8966352 | 3 | Domus de Maria |
| ADS3_12 | 8,87175616 | 38,8966352 | 3 | Domus de Maria |
| ADS3_13 | 8,72881179 | 38,9506036 | 3 | Teulada |
| ADS3_14 | 8,72881179 | 38,9506036 | 3 | Teulada |
| ADS4_1 | 8,55155108 | 39,0787111 | 4 | San Giovanni Suergiu |
| ADS4_2 | 8,55155108 | 39,0787111 | 4 | San Giovanni Suergiu |
| ADS4_3 | 8,64358172 | 39,3232542 | 4 | Domusnovas |
| ADS4_4 | 8,64358172 | 39,3232542 | 4 | Domusnovas |
| ADS4_5 | 8,64358172 | 39,3232542 | 4 | Domusnovas |
| ADS4_6 | 8,64358172 | 39,3232542 | 4 | Domusnovas |
| ADS4_7 | 8,53236147 | 39,597413 | 4 | Arbus |
| ADS4_8 | 8,53236147 | 39,597413 | 4 | Arbus |
| ADS4_9 | 8,47847129 | 39,4511779 | 4 | Fluminimaggiore |
| ADS4_10 | 8,47847129 | 39,4511779 | 4 | Fluminimaggiore |
| ADS5_1 | 8,91262924 | 39,5283459 | 5 | Samassi |
| ADS5_2 | 8,91262924 | 39,5283459 | 5 | Samassi |
| ADS5_3 | 8,97814628 | 39,646891 | 5 | Villanovafranca |
| ADS5_4 | 8,97814628 | 39,646891 | 5 | Villanovafranca |
|    \| ADS5_5 \| \| --- \| | 9,38411234 | 39,6574668 | 5 | Escalaplano |
| ADS5_6 | 9,38411234 | 39,6574668 | 5 | Escalaplano |
| ADS5_7 | 9,48740461 | 39,8478417 | 5 | Gairo |
|    \| ADS5_8 \| \| --- \| | 9,48740461 | 39,8478417 | 5 | Gairo |
| ADS6_1 | 9,56591983 | 39,9458821 | 6 | Villagrande Strisaili |
| ADS6_2 | 9,56591983 | 39,9458821 | 6 | Villagrande Strisaili |
| ADS6_3 | 9,68071829 | 39,898612 | 6 | Tortol |
| ADS6_4 | 9,68071829 | 39,898612 | 6 | Tortol |
|    \| ADS7_1 \| \| --- \| | 9,70876981 | 40,3799189 | 7 | Orosei |
| ADS7_2 | 9,70876981 | 40,3799189 | 7 | Orosei |
| ADS7_3 | 9,72864114 | 40,6359723 | 7 | Posada |
|    \| ADS7_4 \| \| --- \| | 9,72864114 | 40,6359723 | 7 | Posada |
| ADA8_1 | 14,954212 | 40,987362 | 8 | Avellino |
| ADA8_2 | 14,99198 | 41,003938 | 8 | Avellino |
|    \| ADA8_3 \| \| --- \| | 14,958196 | 40,989909 | 8 | Avellino |
| ADA8_4 | 14,958196 | 40,989909 | 8 | Avellino |
| ADE9_1 | 11,50692 | 44,70388 | 9 | Malalbergo |
| ADE9_2 | 11,50692 | 44,70388 | 9 | Malalbergo |
| ADE9_3 | 11,56692 | 44,72358 | 9 | Poggio Renatico |
| ADE9_4 | 11,53692 | 44,71998 | 9 | Malalbergo |
|    \| ADE10_1 \| \| --- \| | 12,83934 | 43,94059 | 10 | Riccione |
| ADE10_2 | 12,64444 | 43,99027 | 10 | Riccione |
| ADE10_3 | 12,64444 | 43,99027 | 10 | Riccione |
|    \| ADE10_4 \| \| --- \| | 12,8227 | 43,9516 | 10 | Pesaro |
| ADE10_5 | 12,8227 | 43,9516 | 10 | Pesaro |
| ADE10_6 | 12,82359 | 43,94729 | 10 | Pesaro |
|    \| ADE10_7 \| \| --- \| | 12,83939 | 43,94038 | 10 | Pesaro |
| ADE10_8 | 12,83939 | 43,94038 | 10 | Pesaro |
| ADE10_9 | 12,83939 | 43,94038 | 10 | Pesaro |
| ADE10_10 | 12,83939 | 43,94038 | 10 | Pesaro |
|    \| ADE10_11 \| \| --- \| | 12,83939 | 43,94038 | 10 | Pesaro |
| ADE10_12 | 12,83939 | 43,94038 | 10 | Pesaro |
| ADE11_1 | 11,3853 | 44,46213 | 11 | Bologna |
|    \| ADE11_2 \| \| --- \| | 11,35915 | 44,45036 | 11 | Bologna |
| ADE11_3 | 11,35915 | 44,45036 | 11 | Bologna |
| ADE11_4 | 11,35915 | 44,45036 | 11 | Bologna |
| ADE12_1 | 11,38917 | 44,46616 | 12 | Bologna |
| ADE12_2 | 11,35851 | 44,43477 | 12 | Pianoro |
| ADE12_3 | 11,35851 | 44,43477 | 12 | Pianoro |
| ADE12_4 | 11,35851 | 44,43477 | 12 | Pianoro |
| ADE13_1 | 11,59685 | 44,27675 | 13 | Borgo Tossignano |
| ADE13_2 | 11,59685 | 44,27675 | 13 | Borgo Tossignano |
| ADE13_3 | 11,28855 | 44,44305 | 13 | Bologna |
| ADE13_4 | 11,6688 | 44,7716 | 13 | Ferrara |
|    \| ADS14_1 \| \| --- \| | 11,3822 | 44,4451 | 14 | San Lazzaro di Savena |
| ADS14_2 | 11,63334 | 44,24887 | 14 | Casola Valsenio |
| ADS14_3 | 11,63334 | 44,24887 | 14 | Casola Valsenio |
|    \| ADS14_4 \| \| --- \| | 11,1583 | 44,41416 | 14 | Monte San Pietro |
