# Supplementary material for: The use of MSAP reveals epigenetic diversity of the invasive clonal populations of Arundo donax L
Source: PLoS One. 2019 Apr 9;14(4):e0215096. doi: 10.1371/journal.pone.0215096 (PMC6456200; doi:10.1371/journal.pone.0215096)
Supplement: S1 Document — (PDF) [file pone.0215096.s006.pdf]

Job icy-smoke-8e39

This output file was generated at:

2019-Mar-18 06:36:33 PDT

This document is not permanent. It will automatically be removed from the server in seven (7) days. Please save or print it for your records. If images are missing, try reloading; this sometimes happens under heavy server load.

Single file archive including this page, all images, all clumpp files: download. [.tar.gz]

L(K)

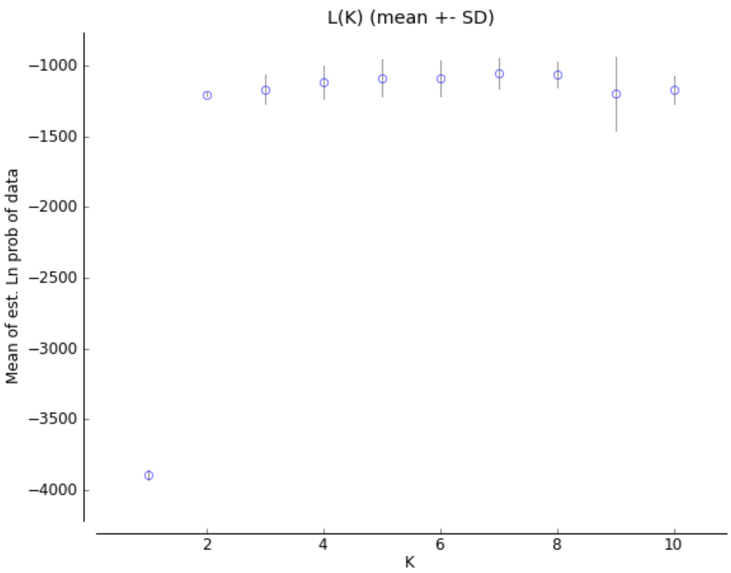

L(K): pdf eps

Clumpp files

K = 1 Clumpp indfile

K = 2 Clumpp indfile

K = 3 Clumpp indfile

K = 4 Clumpp indfile

K = 5 Clumpp indfile

K = 6 Clumpp indfile

K = 7 Clumpp indfile

K = 8 Clumpp indfile

K = 9 Clumpp indfile

K = 10 Clumpp indfile

Evanno method

*Evanno et al., 2005. Molecular Ecology 14, 2611 - 2620. How are we calculating this? Look at the FAQ.

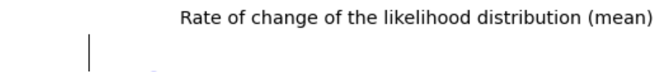

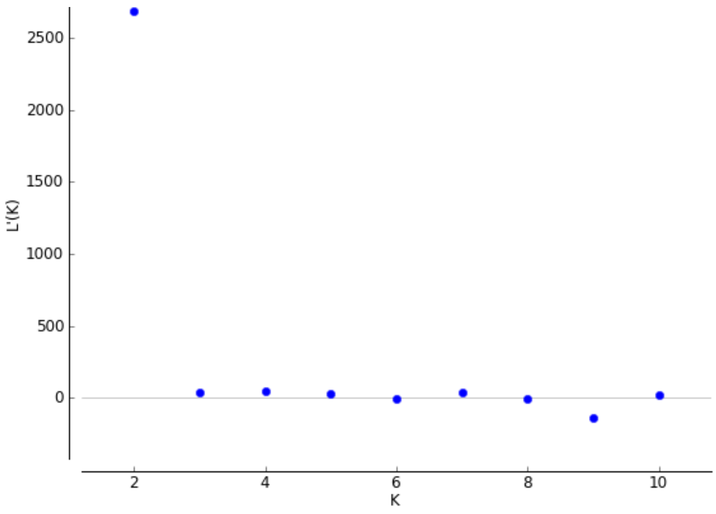

L'(K): pdf eps

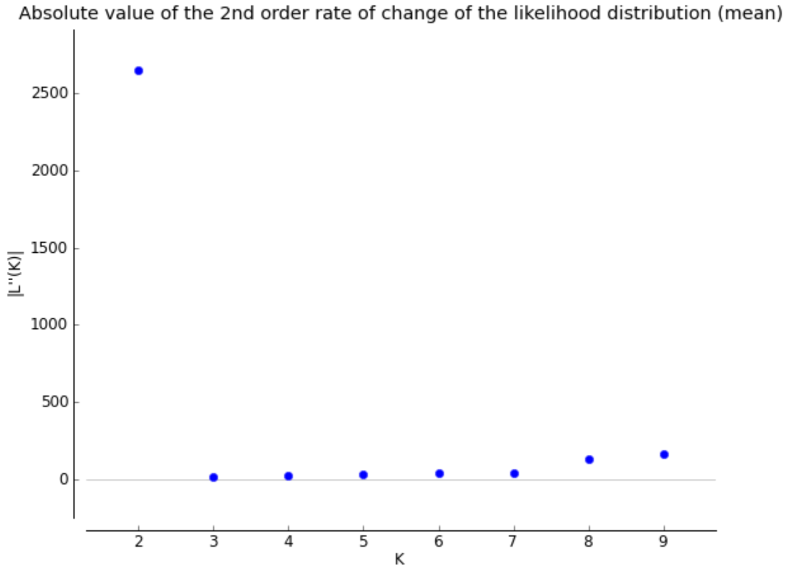

|L''(K)|: pdf eps

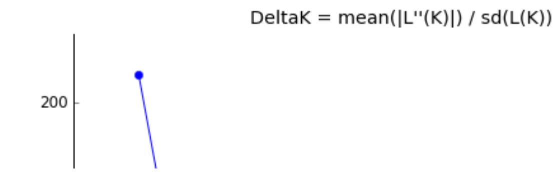

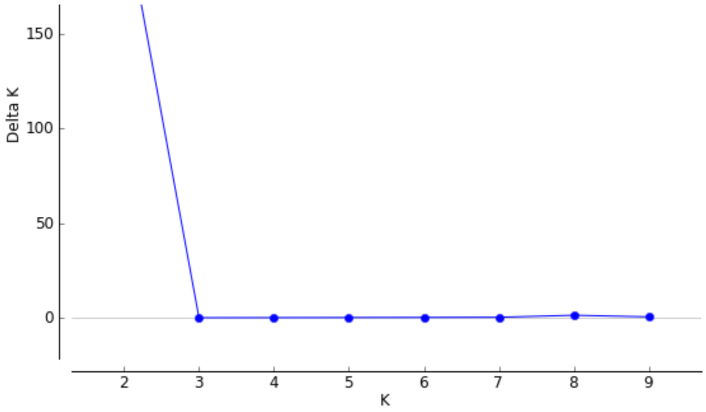

Delta K: pdf eps

The Evanno table output is also available as a tab-delimited text file (for use with Excel) here.

K	Reps	Mean LnP(K)	Stdev LnP(K)	Ln'(K)	|Ln''(K)|	Delta K
1	5	-3891.360000	35.464884	—	—	—
2	5	-1206.460000	12.335031	2684.900000	2648.520000	214.715302
3	5	-1170.080000	104.282079	36.380000	16.080000	0.154197
4	5	-1117.620000	116.287003	52.460000	22.940000	0.197271
5	5	-1088.100000	127.204501	29.520000	32.420000	0.254865
6	5	-1091.000000	122.135908	-2.900000	38.240000	0.313094
7	5	-1055.660000	106.496962	35.340000	41.940000	0.393814
8	5	-1062.260000	90.474599	-6.600000	130.460000	1.441952
9	5	-1199.320000	261.183215	-137.060000	161.513333	0.618391
10	6	-1174.866667	99.199872	24.453333	—	—

Raw STRUCTURE output

The raw STRUCTURE output is also available as a tab-delimited text file (for use with Excel) here.

File name	Run #	K	Est. Ln prob. of data	Mean value of Ln likelihood	Variance of Ln likelihood
Results1000-10000_run_6_f	6	1	-3936.4	-3382.8	1107.1
Results1000-10000_run_5_f	5	1	-3868.1	-3385.6	964.9
Results1000-10000_run_2_f	2	1	-3906.9	-3387.7	1038.5
Results1000-10000_run_4_f	4	1	-3845.1	-3386.9	916.4
Results1000-10000_run_3_f	3	1	-3900.3	-3385.4	1029.9
Results1000-10000_run_9_f	9	2	-1198.3	-957.2	482.2
Results1000-10000_run_10_f	10	2	-1202.7	-958.7	488.1
Results1000-10000_run_11_f	11	2	-1198.0	-956.5	483.0
Results1000-10000_run_8_f	8	2	-1227.8	-958.6	538.5
Results1000-10000_run_7_f	7	2	-1205.5	-956.4	498.1
Results1000-10000_run_13_f	13	3	-1209.9	-957.0	505.8
Results1000-10000_run_14_f	14	3	-1215.7	-959.9	511.6
Results1000-10000_run_16_f	16	3	-1196.7	-957.0	479.4
Results1000-10000_run_15_f	15	3	-1242.2	-957.7	568.9
Results1000-10000_run_12_f	12	3	-985.9	-766.3	439.3
Results1000-10000_run_18_f	18	4	-994.5	-767.7	453.5
Results1000-10000_run_19_f	19	4	-987.0	-766.5	441.1
Results1000-	[illegible]	[illegible]	[illegible]	[illegible]	[illegible]

10000_run_17_f	17	4	-1209.8	-960.7	498.1
Results1000-10000_run_20_f	20	4	-1211.1	-961.0	500.0
Results1000-10000_run_21_f	21	4	-1185.7	-957.3	456.7
Results1000-10000_run_25_f	25	5	-997.7	-773.5	448.4
Results1000-10000_run_23_f	23	5	-1222.5	-965.9	513.3
Results1000-10000_run_24_f	24	5	-990.0	-771.5	436.9
Results1000-10000_run_22_f	22	5	-998.1	-769.6	456.9
Results1000-10000_run_26_f	26	5	-1232.2	-967.2	529.9
Results1000-10000_run_27_f	27	6	-992.5	-778.3	428.3
Results1000-10000_run_29_f	29	6	-1212.1	-969.5	485.2
Results1000-10000_run_30_f	30	6	-1236.5	-970.0	533.0
Results1000-10000_run_31_f	31	6	-1008.0	-770.3	475.4
Results1000-10000_run_28_f	28	6	-1005.9	-772.7	466.3
Results1000-10000_run_36_f	36	7	-1005.8	-780.3	450.9
Results1000-10000_run_35_f	35	7	-1001.7	-780.5	442.4
Results1000-10000_run_33_f	33	7	-1007.8	-785.2	445.2
Results1000-10000_run_34_f	34	7	-1245.9	-974.2	543.3
Results1000-10000_run_32_f	32	7	-1017.1	-782.1	470.0
Results1000-10000_run_41_f	41	8	-1020.3	-784.7	471.2
Results1000-10000_run_39_f	39	8	-1222.1	-982.1	480.0
Results1000-10000_run_37_f	37	8	-1046.0	-792.8	506.4
Results1000-10000_run_40_f	40	8	-1009.8	-789.3	441.1
Results1000-10000_run_38_f	38	8	-1013.1	-785.5	455.1
Results1000-10000_run_42_f	42	9	-1050.0	-796.9	506.2
Results1000-10000_run_45_f	45	9	-1021.7	-791.2	460.9
Results1000-10000_run_44_f	44	9	-1247.5	-797.9	899.2
Results1000-10000_run_46_f	46	9	-1636.9	-951.3	1371.2
Results1000-10000_run_43_f	43	9	-1040.5	-789.1	502.7
Results1000-10000_run_51_f	51	10	-1070.0	-801.7	536.7
Results1000-10000_run_50_f	50	10	-1265.8	-989.7	552.2
Results1000-10000_run_1_f	1	10	-1114.8	-740.5	748.6
Results1000-10000_run_47_f	47	10	-1269.2	-995.4	547.6
Results1000-10000_run_48_f	48	10	-1257.5	-995.2	524.5
Results1000-10000_run_49_f	49	10	-1071.9	-806.5	530.8

CITATION

Earl, Dent A. and vonHoldt, Bridgett M. (2012)

```
STRUCTURE HARVESTER: a website and program for visualizing
STRUCTURE output and implementing the Evanno method.
Conservation Genetics Resources vol. 4 (2) pp. 359-361 doi: 10.1007/s12686-011-9548-7
Core version: vA.2 July 2014
Plot version: vA.1 November 2012
Web version: v0.6.94 July 2014
```

© Dent Earl 2007-2014
