# Supplementary material for: The use of MSAP reveals epigenetic diversity of the invasive clonal populations of Arundo donax L
Source: PLoS One. 2019 Apr 9;14(4):e0215096. doi: 10.1371/journal.pone.0215096 (PMC6456200; doi:10.1371/journal.pone.0215096)
Supplement: S2 Document — (PDF) [file pone.0215096.s007.pdf]

Job muddy-snowflake-ee79

This output file was generated at:

2019-Mar-18 06:38:39 PDT

This document is not permanent. It will automatically be removed from the server in seven (7) days. Please save or print it for your records. If images are missing, try reloading; this sometimes happens under heavy server load.

Single file archive including this page, all images, all clumpp files: download. [.tar.gz]

L(K)

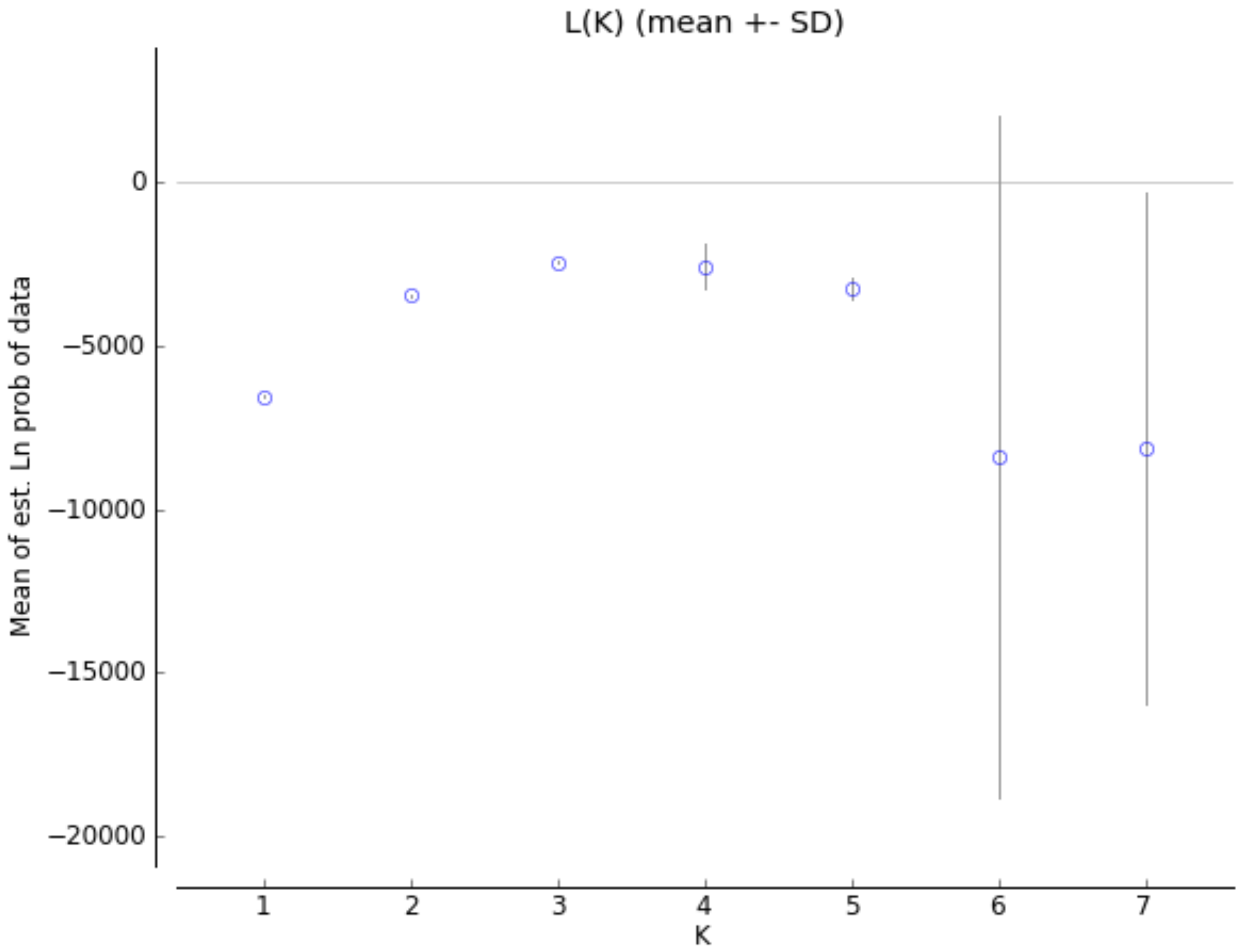

L(K): pdf eps

Clumpp files

K = 1 Clumpp indfile

K = 2 Clumpp indfile

K = 3 Clumpp indfile

K = 4 Clumpp indfile

K = 5 Clumpp indfile

K = 6 Clumpp indfile

K = 7 Clumpp indfile

Evanno method

*Evanno et al., 2005. Molecular Ecology 14, 2611 - 2620. How are we calculating this? Look at the FAQ.

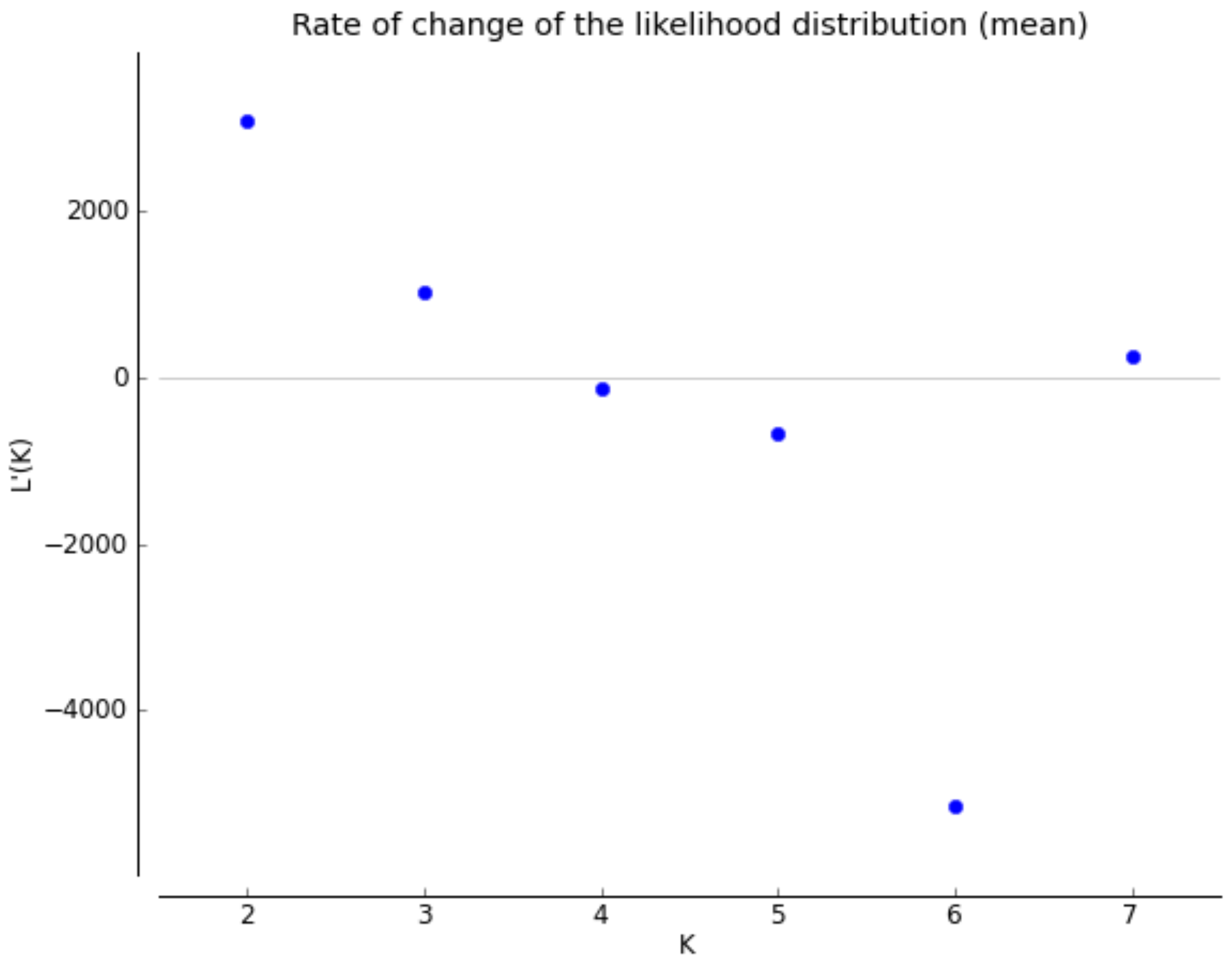

L'(K): pdf eps

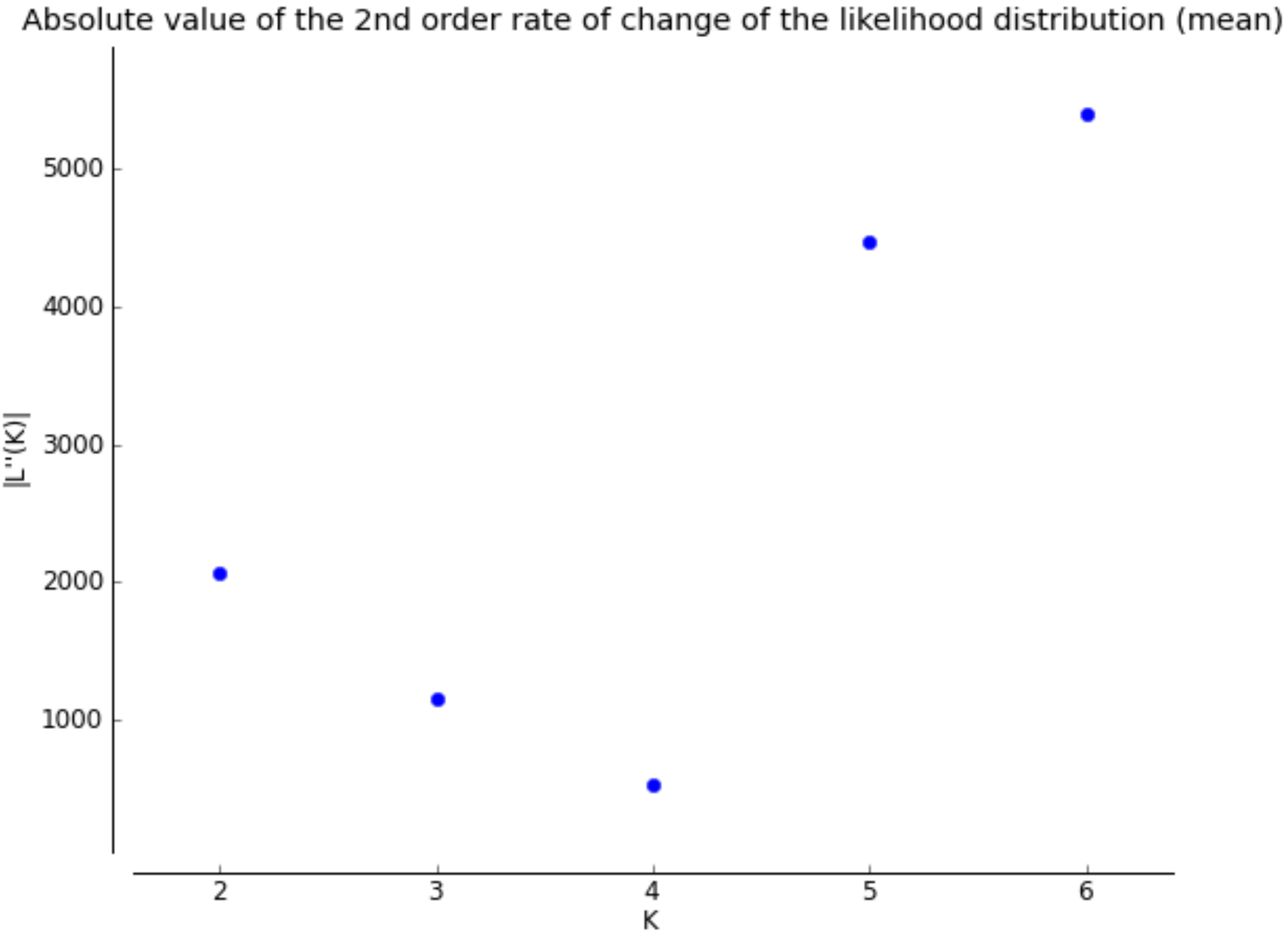
Absolute value of the 2nd order rate of change of the likelihood distribution (mean)
|L''(K)|
5000
4000
3000
2000
1000
2
3
4
5
6
K

|L''(K)|: pdf eps

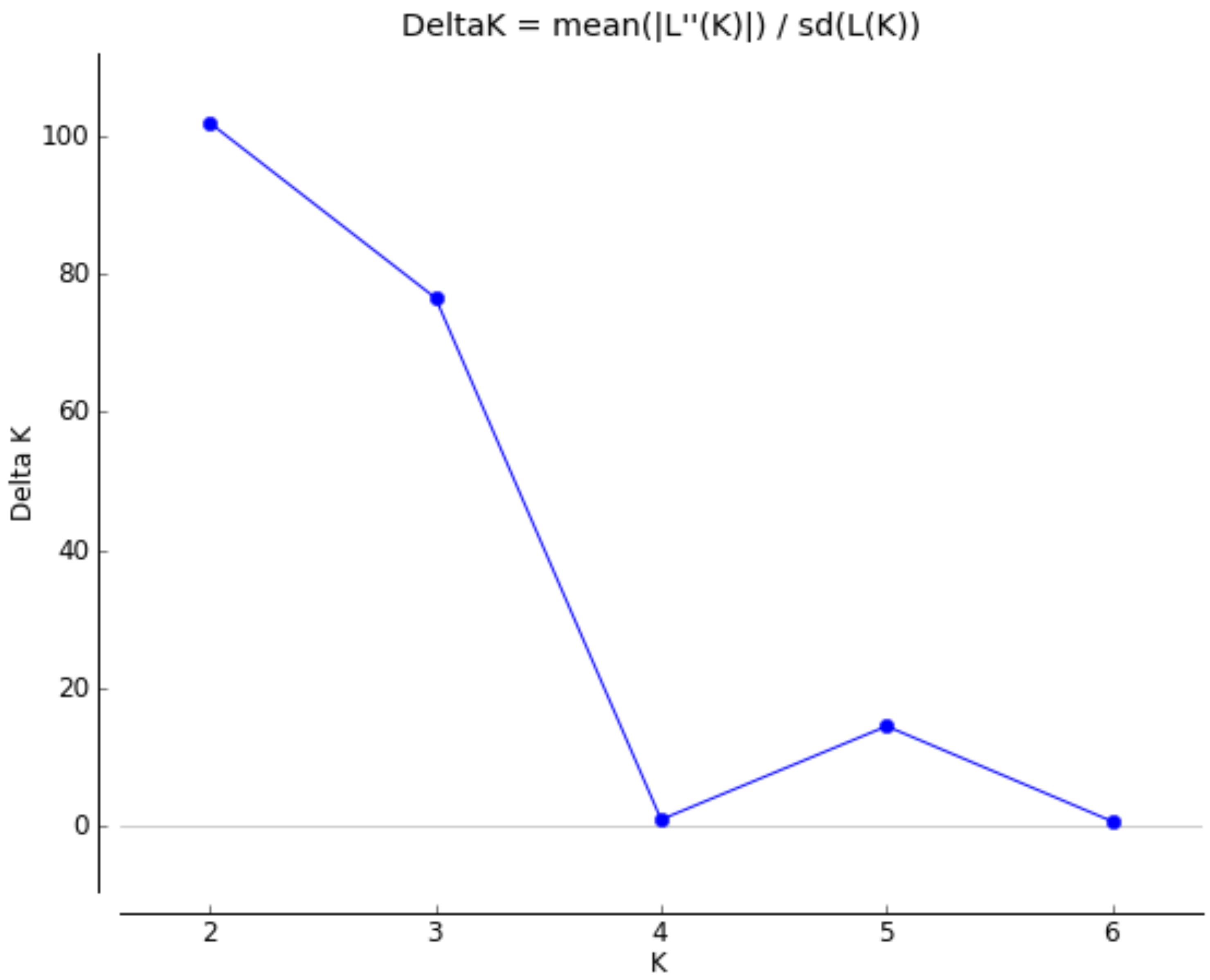

Delta K: pdf eps

The Evanno table output is also available as a tab-delimited text file (for use with Excel) here.

K	Reps	Mean LnP(K)	Stdev LnP(K)	Ln'(K)	|Ln''(K)|	Delta K
1	5	-6531.880000	39.533998	—	—	—
2	5	-3450.260000	20.271482	3081.620000	2067.760000	102.003394
3	5	-2436.400000	15.060710	1013.860000	1153.320000	76.578061
4	5	-2575.860000	677.617486	-139.460000	528.840000	0.780440
5	5	-3244.160000	311.890394	-668.300000	4476.140000	14.351644
6	5	-8388.600000	10432.727934	-5144.440000	5396.790000	0.517294
7	6	-8136.250000	7830.025434	252.350000	—	—

Raw STRUCTURE output

The raw STRUCTURE output is also available as a tab-delimited text file (for use with Excel) here.

File name	Run #	K	Est. Ln prob. of data	Mean value of Ln likelihood	Variance of Ln likelihood
Results1000-10000_run_6_f	6	1	-6570.7	-5605.7	1930.0
Results1000-	5	1	-6538.2	-5601.4	1873.6

10000_run_5_f					
Results1000-10000_run_2_f	2	1	-6535.2	-5602.0	1866.4
Results1000-10000_run_4_f	4	1	-6465.7	-5601.6	1728.2
Results1000-10000_run_3_f	3	1	-6549.6	-5603.4	1892.4
Results1000-10000_run_9_f	9	2	-3463.6	-2864.3	1198.6
Results1000-10000_run_10_f	10	2	-3451.4	-2865.3	1172.2
Results1000-10000_run_11_f	11	2	-3424.5	-2864.1	1120.8
Results1000-10000_run_8_f	8	2	-3436.7	-2861.6	1150.3
Results1000-10000_run_7_f	7	2	-3475.1	-2866.5	1217.2
Results1000-10000_run_13_f	13	3	-2438.1	-1920.1	1036.1
Results1000-10000_run_14_f	14	3	-2449.6	-1921.3	1056.6
Results1000-10000_run_16_f	16	3	-2429.1	-1921.3	1015.6
Results1000-10000_run_15_f	15	3	-2450.6	-1923.1	1055.0
Results1000-10000_run_12_f	12	3	-2414.6	-1919.3	990.7
Results1000-10000_run_18_f	18	4	-2029.6	-1602.1	855.1
Results1000-10000_run_19_f	19	4	-3749.7	-1815.2	3869.0
Results1000-10000_run_17_f	17	4	-2274.8	-1776.3	997.0
Results1000-10000_run_20_f	20	4	-2503.2	-1734.8	1536.7
Results1000-10000_run_21_f	21	4	-2322.0	-1784.1	1075.8
Results1000-10000_run_25_f	25	5	-3630.1	-1705.8	3848.6
Results1000-10000_run_23_f	23	5	-3500.3	-1804.5	3391.8
Results1000-10000_run_24_f	24	5	-3187.3	-1798.7	2777.2
Results1000-10000_run_22_f	22	5	-2945.6	-2261.8	1367.8
Results1000-					

10000_run_26_f	26	5	-2957.5	-1800.9	2313.1
Results1000-10000_run_27_f	27	6	-3968.9	-1829.0	4279.7
Results1000-10000_run_29_f	29	6	-27029.0	-1681.3	50695.4
Results1000-10000_run_30_f	30	6	-4400.7	-1850.7	5100.0
Results1000-10000_run_31_f	31	6	-3507.3	-1499.7	4015.3
Results1000-10000_run_28_f	28	6	-3037.1	-1800.1	2474.1
Results1000-10000_run_36_f	36	7	-4237.6	-1834.0	4807.2
Results1000-10000_run_35_f	35	7	-4207.2	-1862.5	4689.4
Results1000-10000_run_1_f	1	7	-3814.1	-1821.1	3986.1
Results1000-10000_run_33_f	33	7	-23617.5	-1669.1	43896.8
Results1000-10000_run_34_f	34	7	-4020.0	-1513.7	5012.6
Results1000-10000_run_32_f	32	7	-8921.1	-1672.3	14497.5

CITATION

```
Earl, Dent A. and vonHoldt, Bridgett M. (2012)
STRUCTURE HARVESTER: a website and program for visualizing
STRUCTURE output and implementing the Evanno method.
Conservation Genetics Resources vol. 4 (2) pp. 359-361 doi: 10.1007/s12686-011-9548-7
Core version: vA.2 July 2014
Plot version: vA.1 November 2012
Web version: v0.6.94 July 2014
```

© Dent Earl 2007-2014
