# Supplementary material for: The use of MSAP reveals epigenetic diversity of the invasive clonal populations of Arundo donax L
Source: PLoS One. 2019 Apr 9;14(4):e0215096. doi: 10.1371/journal.pone.0215096 (PMC6456200; doi:10.1371/journal.pone.0215096)
Supplement: S3 Document — (PDF) [file pone.0215096.s008.pdf]

Job frosty-rain-49a3

This output file was generated at:

2019-Mar-18 06:39:58 PDT

This document is not permanent. It will automatically be removed from the server in seven (7) days. Please save or print it for your records. If images are missing, try reloading; this sometimes happens under heavy server load.

Single file archive including this page, all images, all clumpp files: download. [.tar.gz]

L(K)

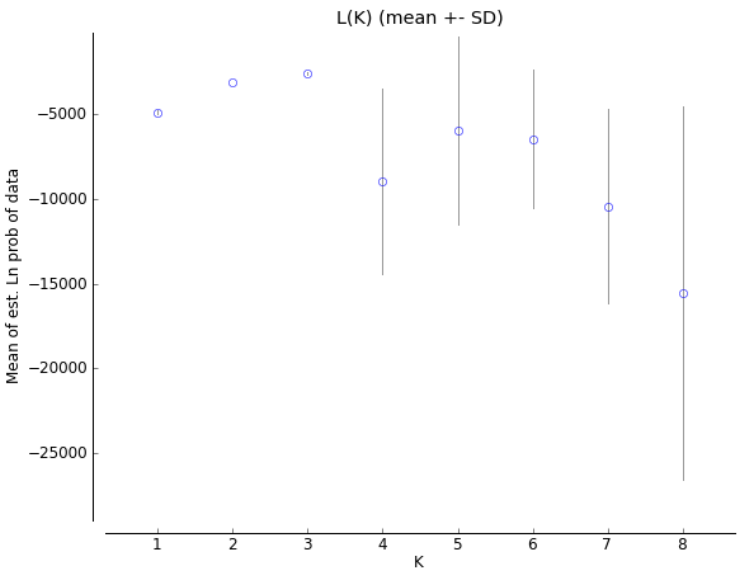

L(K): pdf eps

Clumpp files

K = 1 Clumpp indfile

K = 2 Clumpp indfile

K = 3 Clumpp indfile

K = 4 Clumpp indfile

K = 5 Clumpp indfile

K = 6 Clumpp indfile

K = 7 Clumpp indfile

K = 8 Clumpp indfile

Evanno method

*Evanno et al., 2005. Molecular Ecology 14, 2611 - 2620. How are we calculating this? Look at the FAQ.

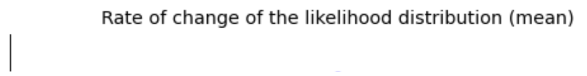

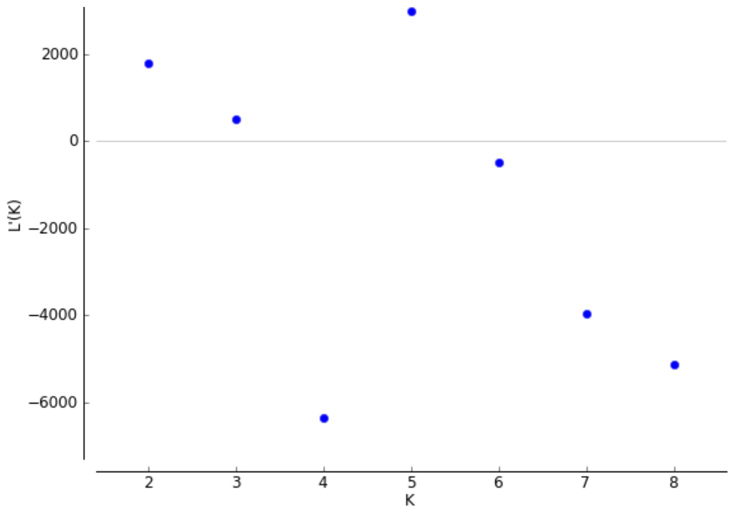

L'(K): pdf eps

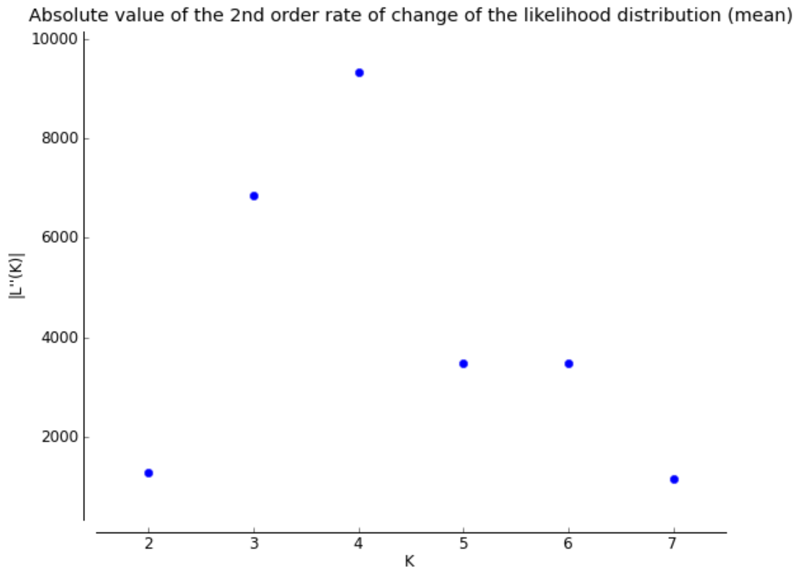

|L''(K)|: pdf eps

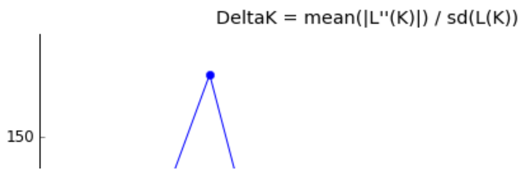

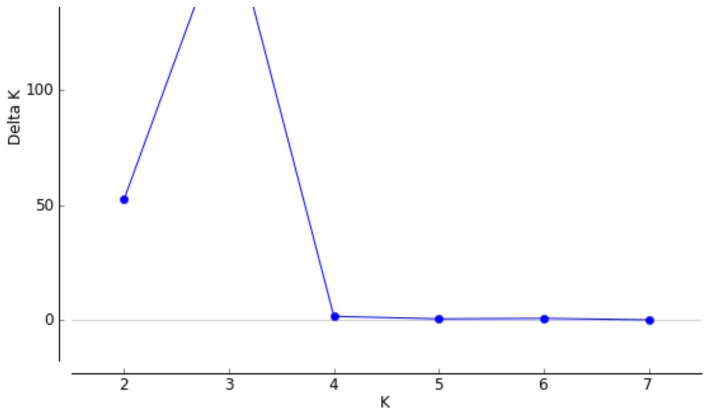

Delta K: pdf eps

The Evanno table output is also available as a tab-delimited text file (for use with Excel) here.

K	Reps	Mean LnP(K)	Stdev LnP(K)	Ln'(K)	|Ln''(K)|	Delta K
1	10	-4917.800000	106.862050	—	—	—
2	10	-3129.340000	24.239509	1788.460000	1273.760000	52.548920
3	10	-2614.640000	38.807078	514.700000	6864.540000	176.888864
4	10	-8964.480000	5467.302233	-6349.840000	9340.680000	1.708462
5	10	-5973.640000	5517.389051	2990.840000	3485.430000	0.631717
6	10	-6468.230000	4100.908499	-494.590000	3468.900000	0.845886
7	10	-10431.720000	5748.485227	-3963.490000	1148.408182	0.199776
8	11	-15543.618182	11008.014977	-5111.898182	—	—

Raw STRUCTURE output

The raw STRUCTURE output is also available as a tab-delimited text file (for use with Excel) here.

File name	Run #	K	Est. Ln prob. of data	Mean value of Ln likelihood	Variance of Ln likelihood
Results1000-10000_run_6_f	6	1	-4810.8	-4392.8	836.0
Results1000-10000_run_9_f	9	1	-4888.2	-4396.6	983.2
Results1000-10000_run_10_f	10	1	-4813.7	-4392.1	843.2
Results1000-10000_run_11_f	11	1	-5045.1	-4411.4	1267.3
Results1000-10000_run_8_f	8	1	-4873.2	-4395.7	955.1
Results1000-10000_run_7_f	7	1	-4992.6	-4404.3	1176.7
Results1000-10000_run_5_f	5	1	-5038.9	-4418.1	1241.6
Results1000-10000_run_2_f	2	1	-4826.3	-4393.0	866.5
Results1000-10000_run_4_f	4	1	-4819.4	-4392.6	853.7
Results1000-10000_run_3_f	3	1	-5069.8	-4412.2	1315.2
Results1000-10000_run_13_f	13	2	-3118.6	-2731.2	774.8
Results1000-10000_run_18_f	18	2	-3076.7	-2731.0	691.5
Results1000-10000_run_14_f	14	2	-3131.1	-2734.3	793.6
Results1000-10000_run_16_f	16	2	-3132.3	-2726.9	810.8
Results1000-10000_run_15_f	15	2	-3150.6	-2740.5	820.1
Results1000-10000_run_12_f	12	2	-3133.3	-2739.0	788.6
Results1000-10000_run_19_f	19	2	-3143.7	-2736.2	815.1
Results1000-10000_run_17_f	17	2	-3132.5	-2732.9	799.2
Results1000-	20	2	-3165.4	-2754.8	821.3

10000_run_20_f	[illegible]	[illegible]	[illegible]	[illegible]	[illegible]
Results1000-10000_run_21_f	21	2	-3109.2	-2730.3	757.8
Results1000-10000_run_25_f	25	3	-2632.2	-2173.5	917.6
Results1000-10000_run_27_f	27	3	-2620.3	-2162.1	916.5
Results1000-10000_run_23_f	23	3	-2615.6	-2172.5	886.2
Results1000-10000_run_29_f	29	3	-2696.6	-2170.4	1052.4
Results1000-10000_run_24_f	24	3	-2548.5	-2168.1	760.9
Results1000-10000_run_22_f	22	3	-2600.3	-2160.6	879.4
Results1000-10000_run_26_f	26	3	-2588.6	-2169.5	838.1
Results1000-10000_run_30_f	30	3	-2587.5	-2167.2	840.7
Results1000-10000_run_31_f	31	3	-2636.2	-2165.3	941.8
Results1000-10000_run_28_f	28	3	-2620.6	-2159.4	922.5
Results1000-10000_run_41_f	41	4	-17605.1	-2033.0	31144.3
Results1000-10000_run_36_f	36	4	-14379.6	-1788.2	25182.9
Results1000-10000_run_35_f	35	4	-2337.1	-1720.4	1233.4
Results1000-10000_run_39_f	39	4	-14555.5	-1895.9	25319.1
Results1000-10000_run_37_f	37	4	-8257.4	-1877.0	12760.7
Results1000-10000_run_40_f	40	4	-3255.6	-2069.6	2371.9
Results1000-10000_run_33_f	33	4	-4835.6	-1810.9	6049.4
Results1000-10000_run_38_f	38	4	-6315.1	-1830.7	8968.7
Results1000-10000_run_34_f	34	4	-5009.4	-1764.7	6489.4
Results1000-10000_run_32_f	32	4	-13094.4	-1800.0	22588.8
Results1000-10000_run_51_f	51	5	-3587.5	-2096.0	2983.1
Results1000-10000_run_42_f	42	5	-3355.9	-1746.3	3219.3
Results1000-10000_run_50_f	50	5	-2551.8	-1701.0	1701.5
Results1000-10000_run_47_f	47	5	-3406.3	-1684.2	3444.2
Results1000-10000_run_48_f	48	5	-13496.7	-1843.6	23306.3
Results1000-10000_run_45_f	45	5	-2372.0	-1711.5	1321.0
Results1000-10000_run_49_f	49	5	-4320.2	-1687.5	5265.5
Results1000-10000_run_44_f	44	5	-18697.1	-1740.0	33914.2
Results1000-10000_run_46_f	46	5	-3286.8	-1596.9	3379.9
Results1000-10000_run_43_f	43	5	-4662.1	-1732.0	5860.3
Results1000-10000_run_52_f	52	6	-2666.9	-1723.0	1887.9
Results1000-10000_run_55_f	55	6	-2602.6	-1714.0	1777.2
Results1000-10000_run_59_f	59	6	-9087.8	-1798.6	14578.4
Results1000-10000_run_56_f	56	6	-11455.4	-1881.5	19147.9
Results1000-10000_run_54_f	54	6	-2387.7	-1589.6	1596.2
Results1000-					

Results1000-10000_run_61_f	61	6	-8679.1	-1700.6	13957.1
Results1000-10000_run_60_f	60	6	-3017.1	-1735.6	2563.0
Results1000-10000_run_58_f	58	6	-6033.8	-1619.4	8828.7
Results1000-10000_run_57_f	57	6	-4921.6	-1593.7	6655.8
Results1000-10000_run_53_f	53	6	-13830.3	-1727.7	24205.2
Results1000-10000_run_67_f	67	7	-18082.3	-1692.1	32780.5
Results1000-10000_run_65_f	65	7	-8748.8	-1713.3	14071.0
Results1000-10000_run_66_f	66	7	-6510.3	-1642.9	9735.0
Results1000-10000_run_68_f	68	7	-6598.0	-1668.0	9860.0
Results1000-10000_run_62_f	62	7	-16141.3	-1746.2	28790.2
Results1000-10000_run_71_f	71	7	-11176.3	-1807.3	18738.1
Results1000-10000_run_63_f	63	7	-7533.4	-1796.1	11474.5
Results1000-10000_run_64_f	64	7	-5899.0	-1710.8	8376.5
Results1000-10000_run_70_f	70	7	-20181.9	-1879.8	36604.1
Results1000-10000_run_69_f	69	7	-3445.9	-1613.8	3664.2
Results1000-10000_run_78_f	78	8	-23531.1	-1876.2	43309.8
Results1000-10000_run_79_f	79	8	-15149.5	-1772.6	26753.8
Results1000-10000_run_1_f	1	8	-2214.4	-1598.5	1231.8
Results1000-10000_run_77_f	77	8	-30754.6	-1790.9	57927.4
Results1000-10000_run_75_f	75	8	-25846.8	-1778.9	48135.8
Results1000-10000_run_80_f	80	8	-22541.1	-1799.9	41482.4
Results1000-10000_run_73_f	73	8	-8072.1	-1632.3	12879.5
Results1000-10000_run_74_f	74	8	-3777.3	-1621.9	4310.7
Results1000-10000_run_76_f	76	8	-8369.8	-1533.7	13672.1
Results1000-10000_run_72_f	72	8	-28358.6	-1596.6	53523.9
Results1000-10000_run_81_f	81	8	-2364.5	-1589.1	1550.8

CITATION

```
Earl, Dent A. and vonHoldt, Bridgett M. (2012)
STRUCTURE HARVESTER: a website and program for visualizing
STRUCTURE output and implementing the Evanno method.
Conservation Genetics Resources vol. 4 (2) pp. 359-361 doi: 10.1007/s12686-011-9548-7
Core version: vA.2 July 2014
Plot version: vA.1 November 2012
Web version: v0.6.94 July 2014
```

© Dent Earl 2007-2014
